# Supplementary material for: The Effect of Neddylation Blockade on Slug-Dependent Cancer Cell Migration Is Regulated by p53 Mutation Status
Source: Cancers (Basel). 2021 Jan 30;13(3):531. doi: 10.3390/cancers13030531 (PMC7866814; doi:10.3390/cancers13030531)
Supplement: Supplementary file 1 [file cancers-13-00531-s001.zip › Cancers supplementary figures.pdf]

cancers

MDPI

Supplementary Materials

The Effect of Neddylation Blockade on Slug-Dependent Cancer Cell Migration is Regulated by p53 Mutation Status

Yelee Kim, Jun Bum Park, Junji Fukuda, Masatoshi Watanabe and Yang-Sook Chun

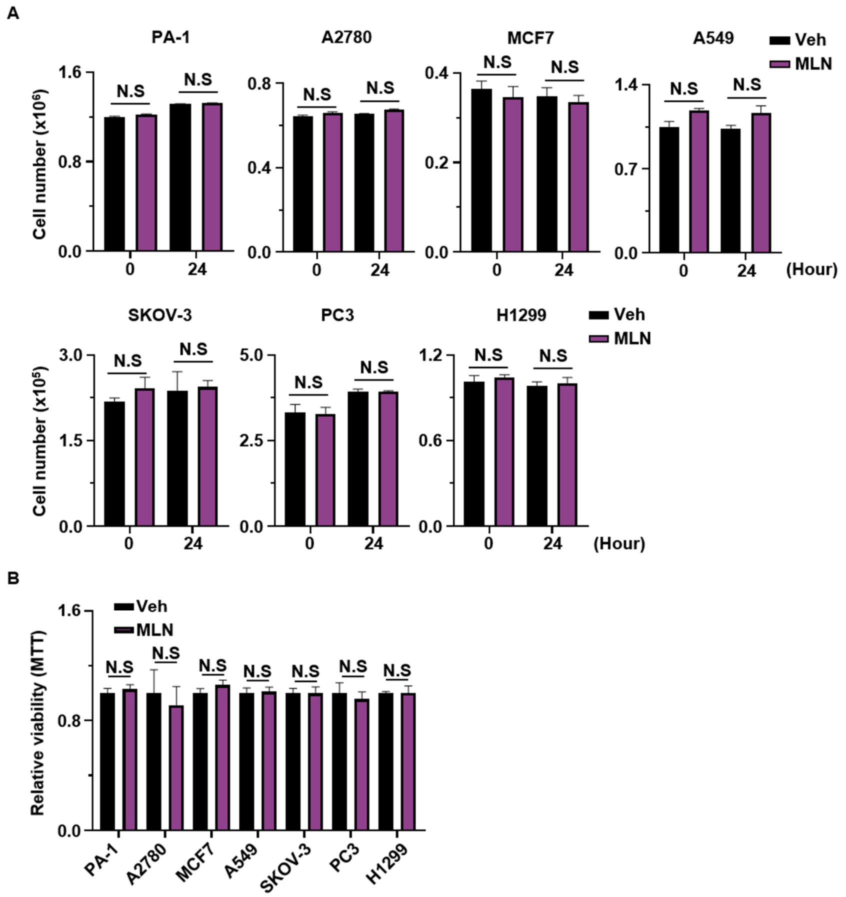

Figure S1. Neddylation blockade does not affect number of cells and cell viability **(A)** The cells were pre-incubated in serum-free media for 1 day then treated with or without 125 nM MLN4924. After 24 h, cell counting was conducted using a hematocytometer. Bars are the means ± standard deviation (*n* = 3). NS, not significant **(B)** After treatment with MLN4924 (125 nM) for 24 h, the cells were incubated with MTT solution to measure the cell viability. Bars represent the means ± standard deviation (*n* = 3). NS, not significant.

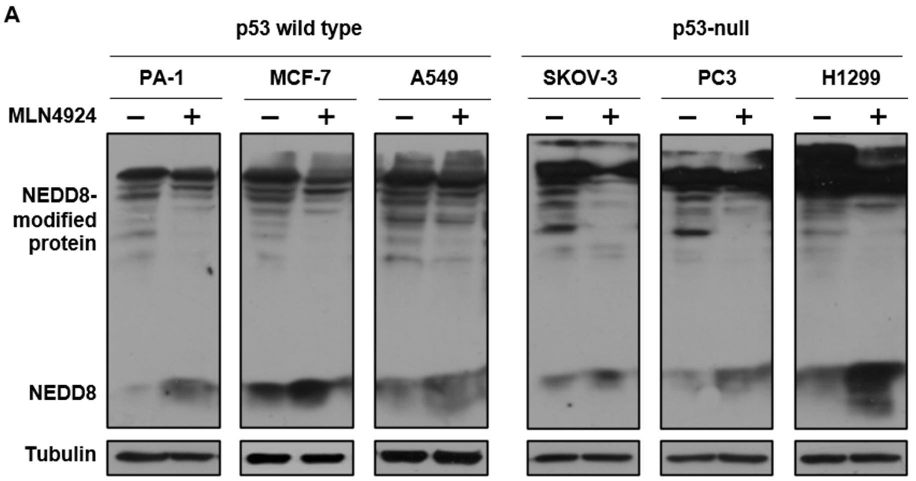

Figure S2. Decreased NEDD8-modified proteins and increased unbound NEDD8 proteins by MLN4924 treatment **(A)** Cancer cells treated with vehicle or MLN4924 were subjected to western blot assay.

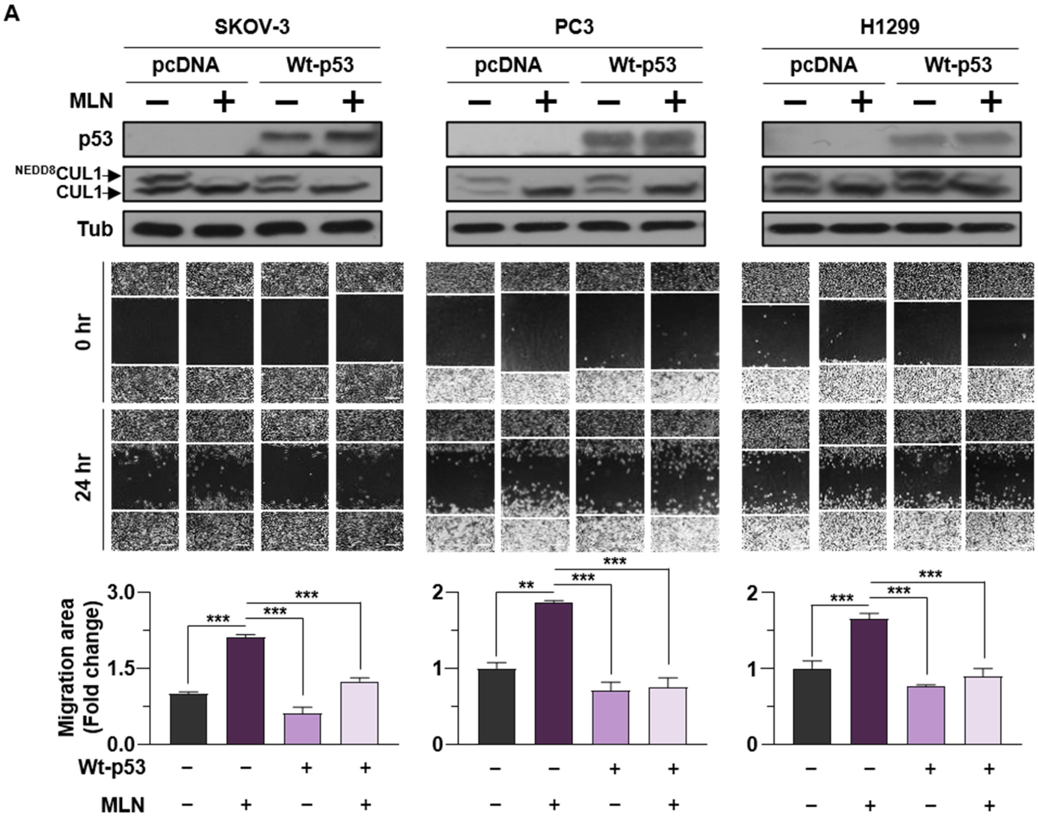

Figure S3. Overexpression of wild-type p53 suppresses MLN4924-induced cell migration in p53-null cancer cells **(A)** SKOV-3, PC3 and H1299 transfected with pcDNA or wild-type p53 and/or treated with MLN4924 (125 nM) were done wound healing assay. Then the cell lysates were subjected to immunoblotting. Scale bar, 200 μm. Whole areas were measured using ImageJ software and data are presented as the means ± standard deviation (n = 3). $^{**}p < 0.01$; $^{***}p < 0.001$; NS, not significant.

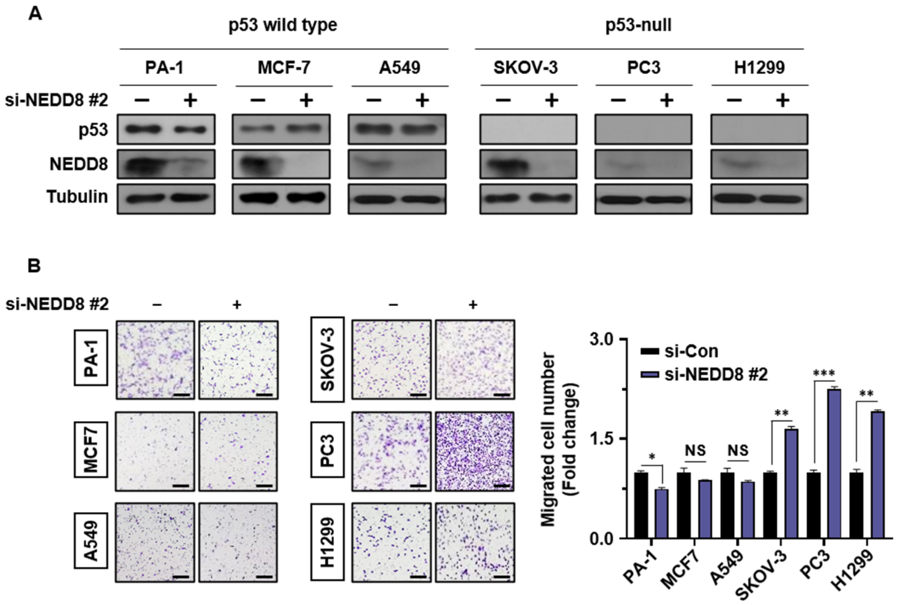

Figure S4. Cancer cell migration with Neddylation blockade using si-NEDD8 #2 **(A,B)** Cancer cells transfected with si-Control or si-NEDD8#2 were subjected to western blot and Transwell assays. The numbers of cells in four randomly chosen fields were counted. Bars represent the means ± standard deviation ($n = 3$). *$p < 0.05$; **$p < 0.01$; ***$p < 0.001$; NS, not significant.

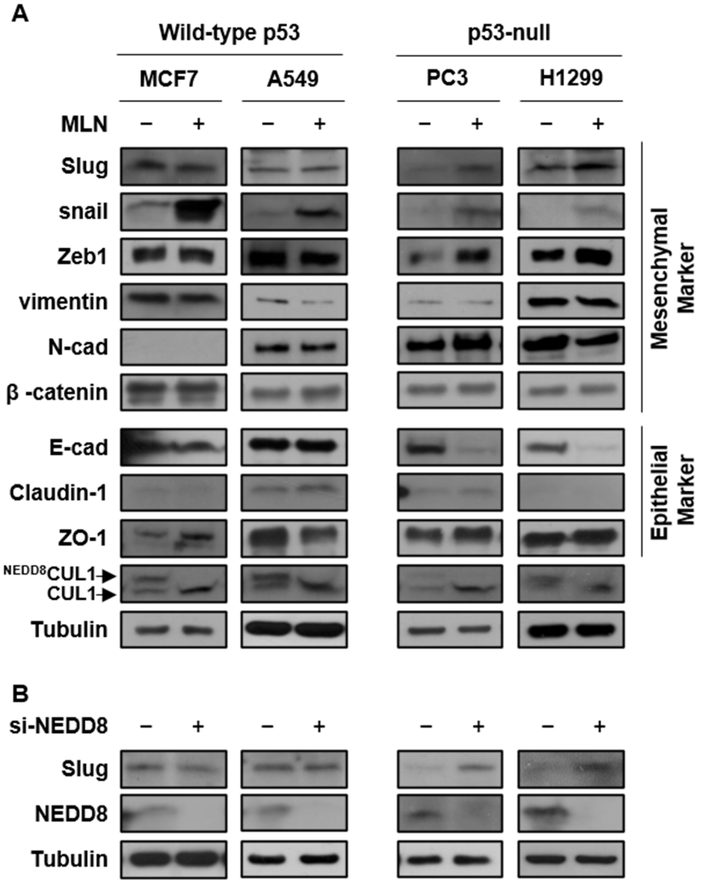

Figure S5. EMT-related markers in different wild-type p53 and p53-null cancer cell lines **(A, B)** Cells were treated with or without MLN4924 (125 nM) or transfected with si-Control or si-NEDD8. The protein levels of representative EMT markers were verified by western blot assay.

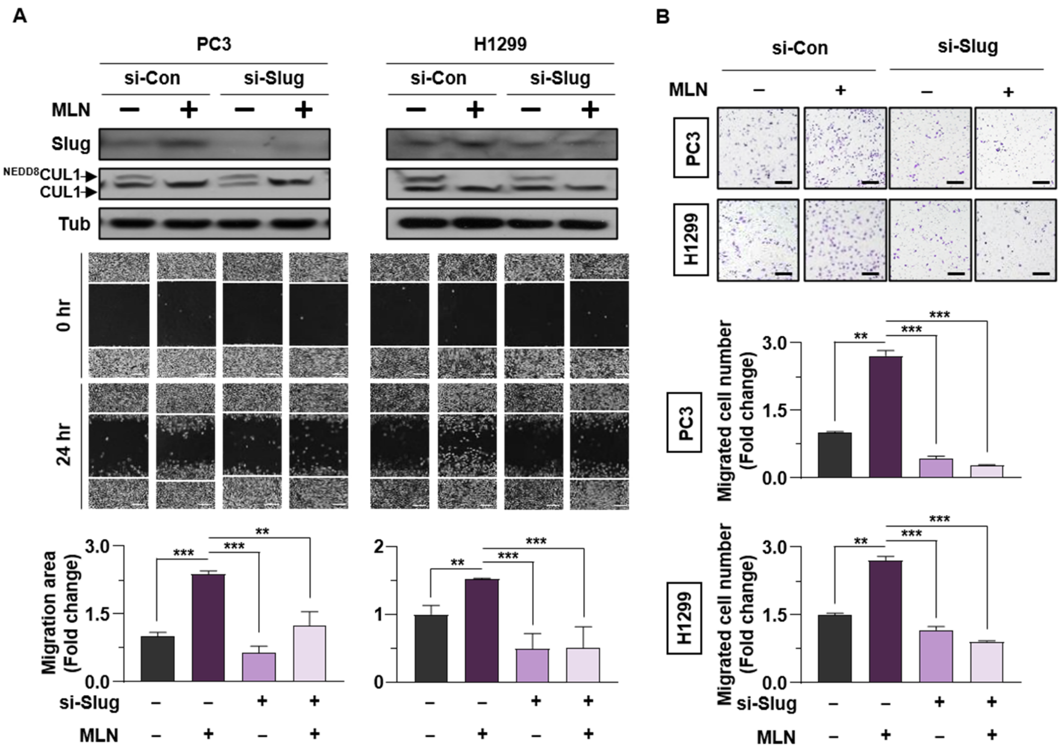

Figure S6. Slug mediates MLN4924-induced EMT in PC3 and H1299 cells **(A)** PC3 and H1299 transfected with si-Control or si-Slug#1 and/or treated with MLN4924 (125 nM) were done wound healing assay. Then the cell lysates were subjected to immunoblotting to verify protein level of Slug and E-cadherin. Scale bar, 200 μm. Whole areas were measured using ImageJ software and data are presented as the means ± standard deviation (n = 3). **(B)** Cells transfected with si-Control or si-Slug#1 and/or treated with MLN4924 (125 nM) were done Transwell assay. The numbers of cells in four randomly chosen fields were counted. $^{**}p < 0.01$; $^{***}p < 0.001$; NS, not significant.

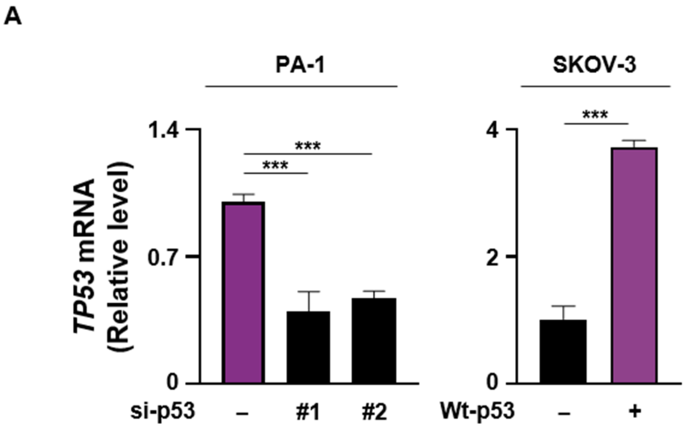

Figure S7. Confirmation of p53 knockdown in PA-1 cells and wild-type p53 overexpression in SKOV-3 cells by RT-qPCR **(A)** PA-1 cells transfected with si-Control, si-p53#1, and si-p53#2, and SKOV-3 cells transfected with pcDNA and wild-type p53 were subjected to RT-qPCR. Data are expressed as means ± standard deviation (n = 3). $^{*}p < 0.01$; $^{***}p < 0.001$; NS, not significant.

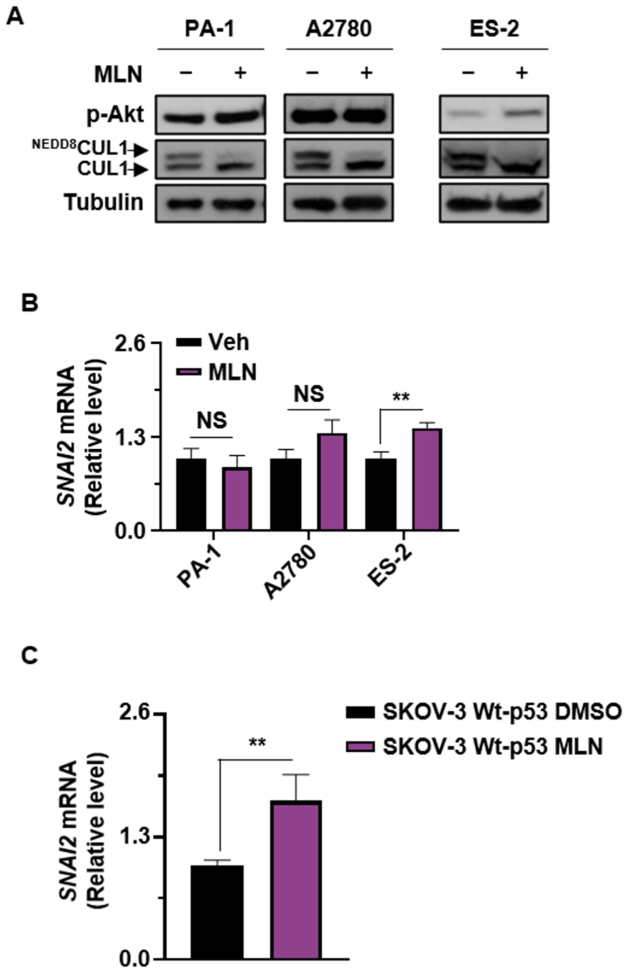

Figure S8. Activation of Akt and Slug expression levels in PA-1, A2780, ES-2, and wild-type p53 expressing-SKOV-3 cells **(A)** PA-1, A2780, and ES-2 cells were treated with vehicle or MLN4924, and then subjected to western blot assays. **(B)** Cells treated with vehicle or MLN4924 were subjected to RT-qPCR. **(C)** SKOV-3 cells transfected with wild-type p53 and treated with or without MLN4924 were subjected to RT-qPCR. Data are expressed as means ± standard deviation (n = 3). **p < 0.01; ***p < 0.001; NS, not significant.

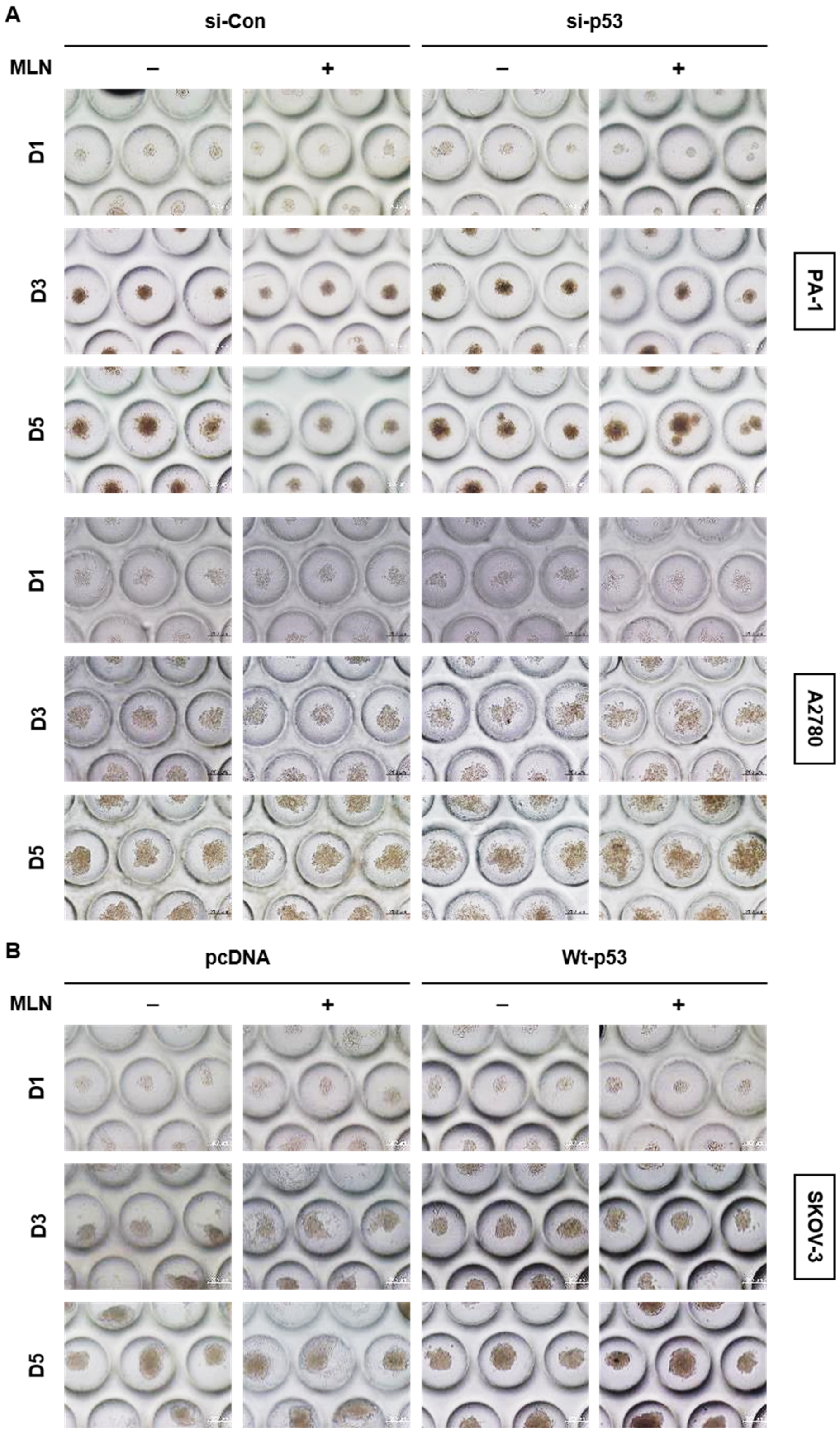

Figure S9. Images of spheroid in PDMS three-dimensional culture chips **(A)** PA-1 and A2780 cells transfected with si-Control or si-p53 were incubated in PDMS 3D chip with culture medium added by vehicle or MLN4924. (**B**) SKOV-3 cells transfected with pcDNA or Wt-p53 were seeded in PDMS 3D culture chip with culture medium added by vehicle or MLN4924. Representative optical microscopy images on day 1, 3, and 5.
