# Supplementary material for: The Effect of Neddylation Blockade on Slug-Dependent Cancer Cell Migration Is Regulated by p53 Mutation Status
Source: Cancers (Basel). 2021 Jan 30;13(3):531. doi: 10.3390/cancers13030531 (PMC7866814; doi:10.3390/cancers13030531)
Supplement: Supplementary file 1 [file cancers-13-00531-s001.zip › Figure S10-uncropped western blot.pdf]

Figure 1A

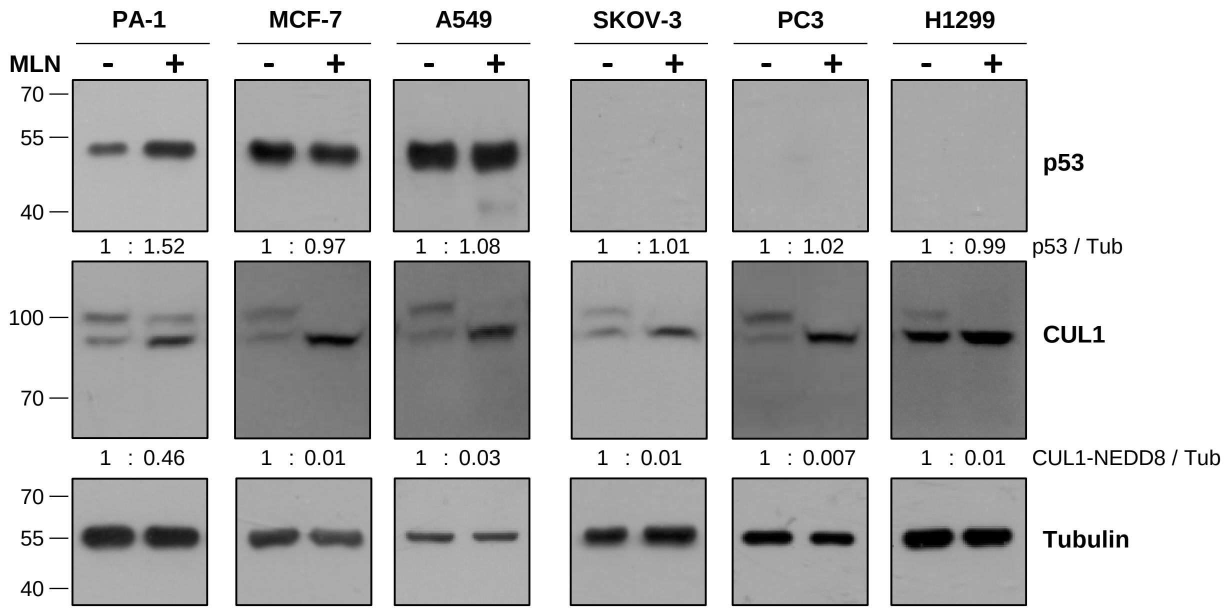

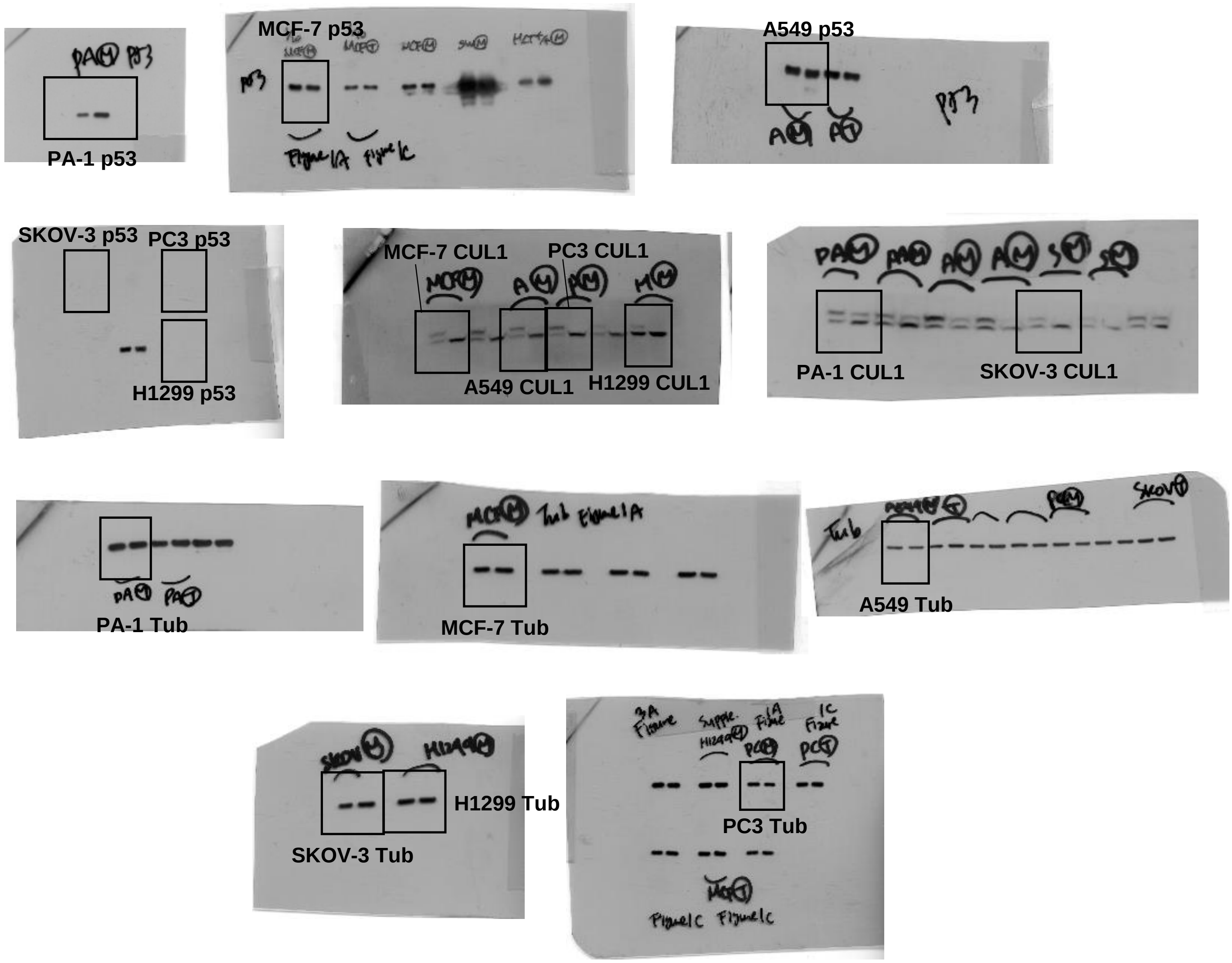

Original blots for western blot analysis shown in Figure 1A.

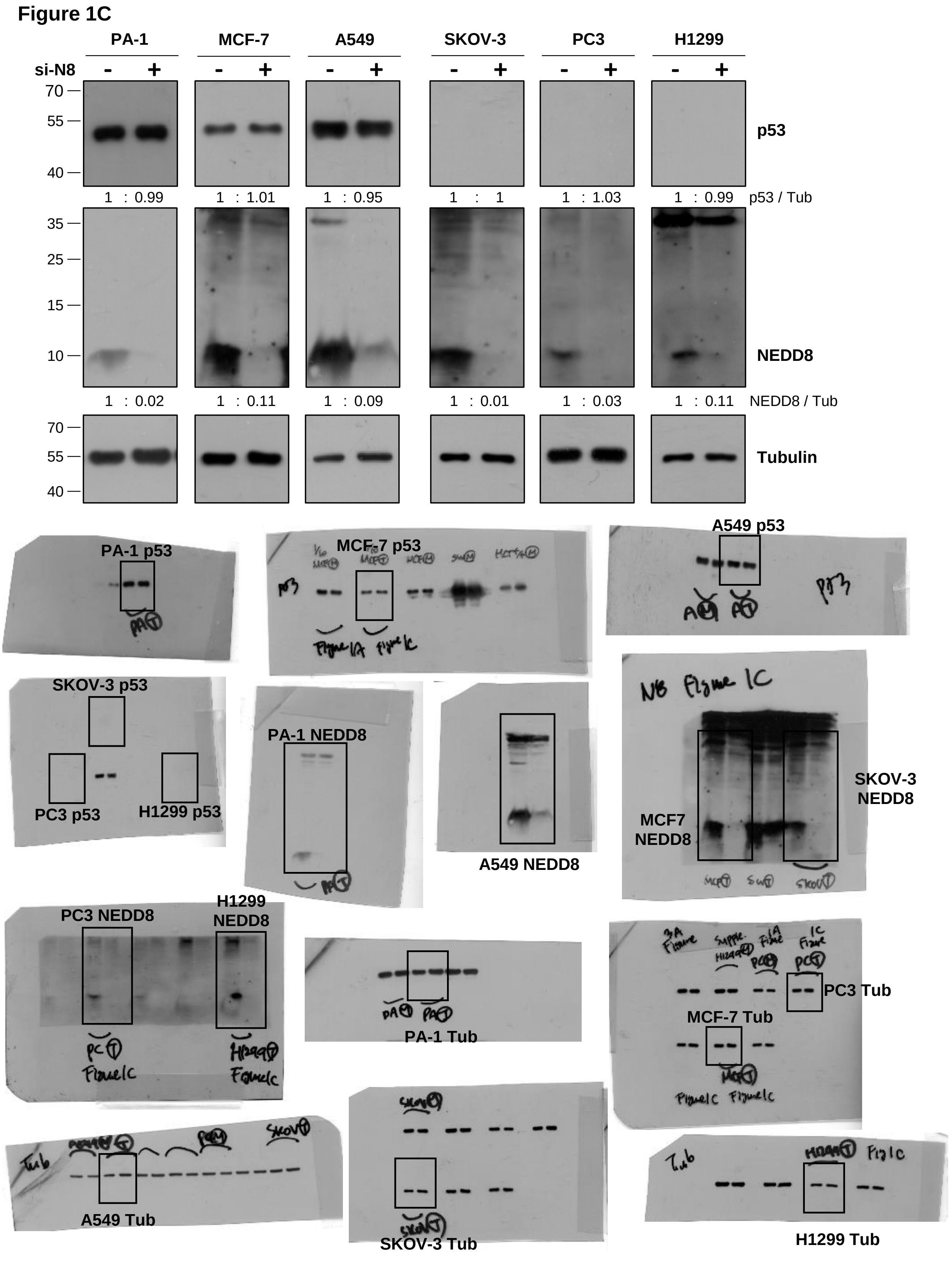

Original blots for western blot analysis shown in Figure 1C.

Figure 3A

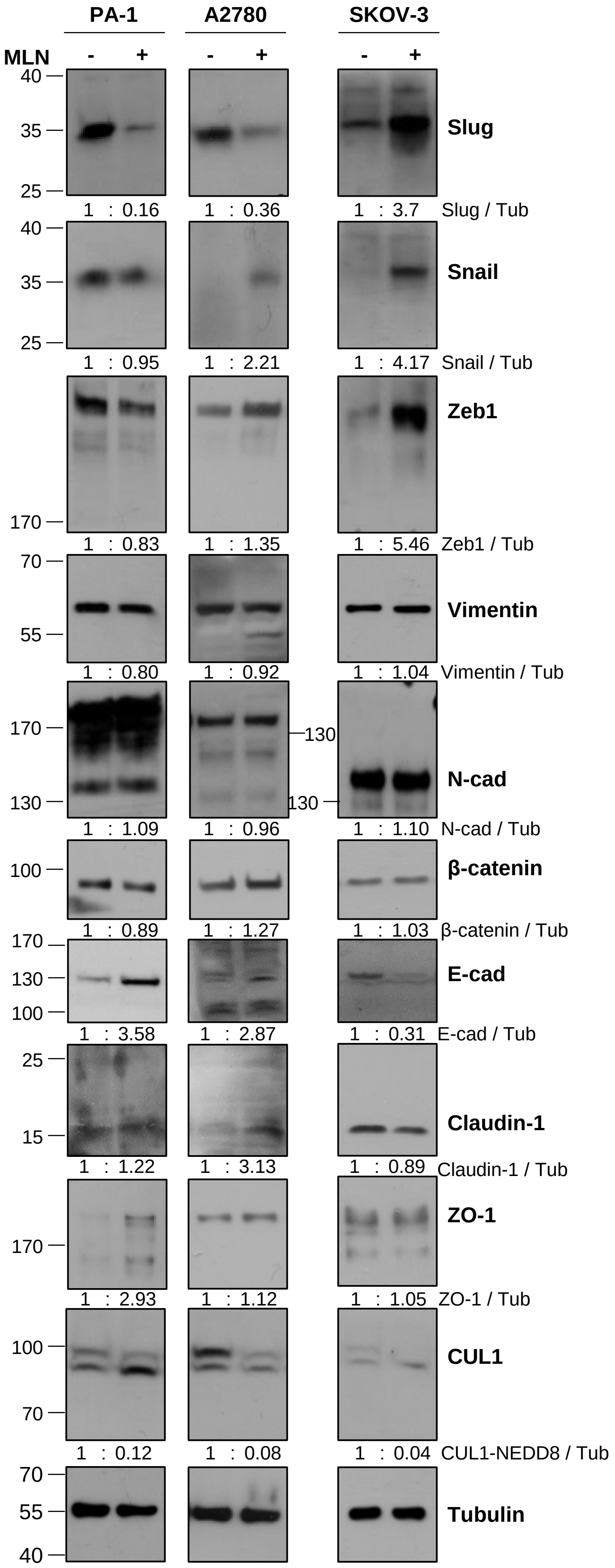

Original blots for western blot analysis shown in Figure 3A

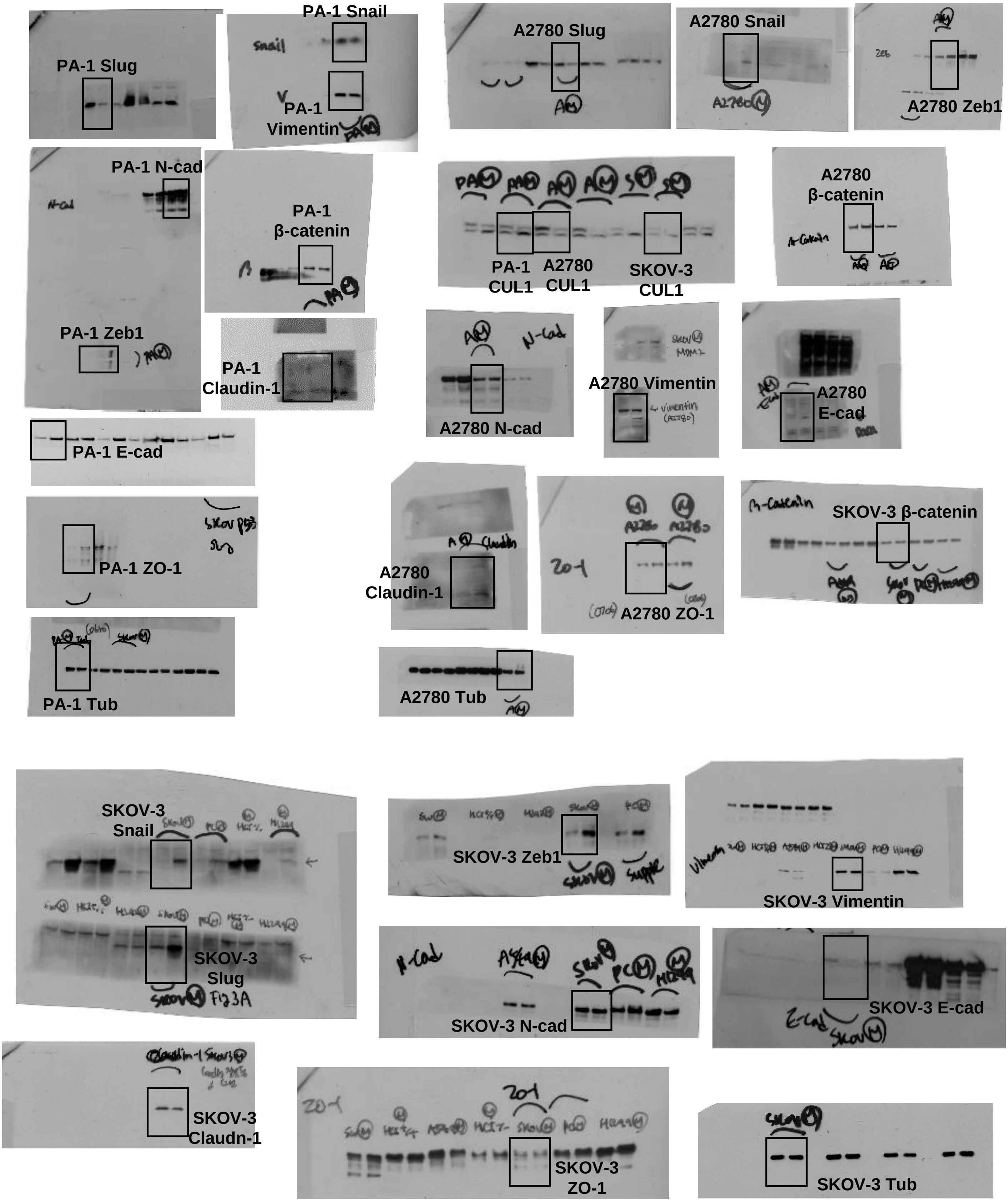

Original blots for western blot analysis shown in Figure 3A

Figure 3B

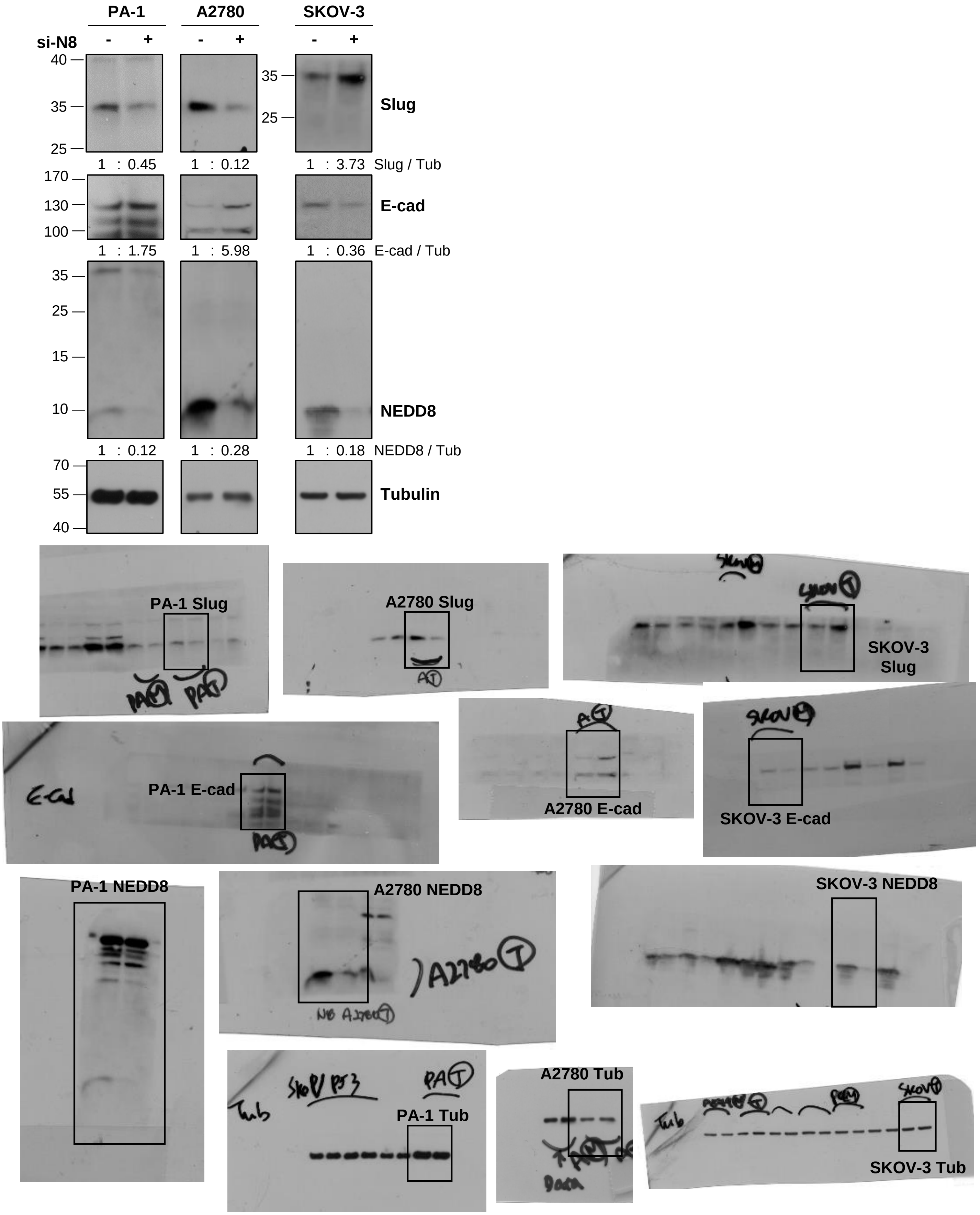

Original blots for western blot analysis shown in Figure 3B

Figure 3E

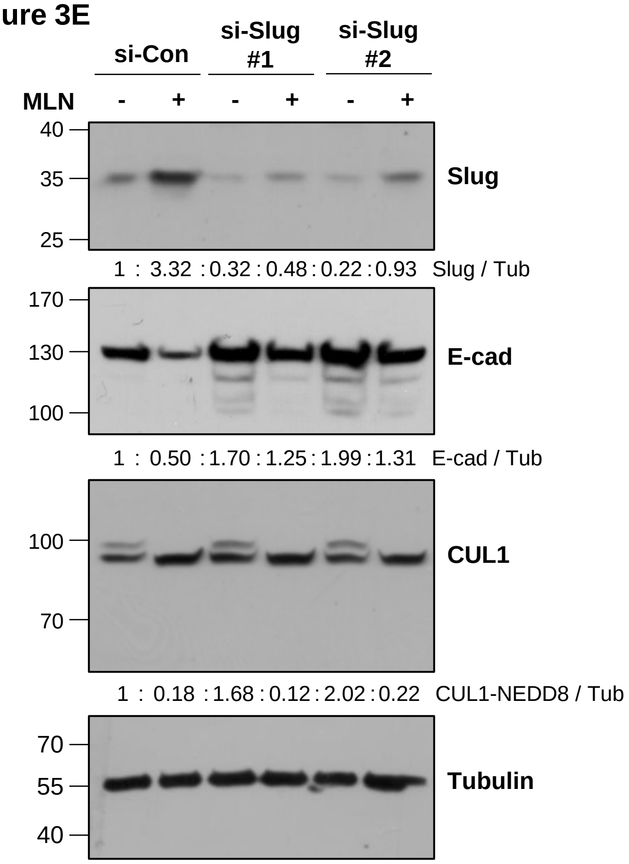

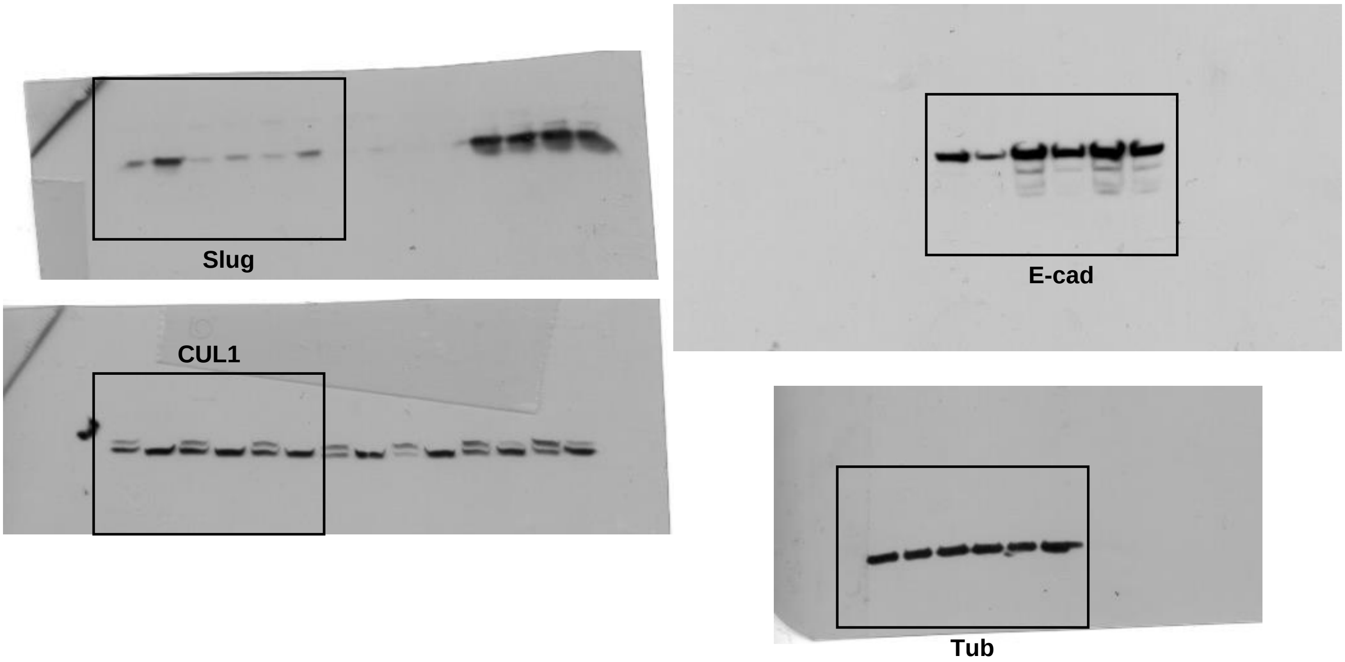

Original blots for western blot analysis shown in Figure 3E

Figure 4A

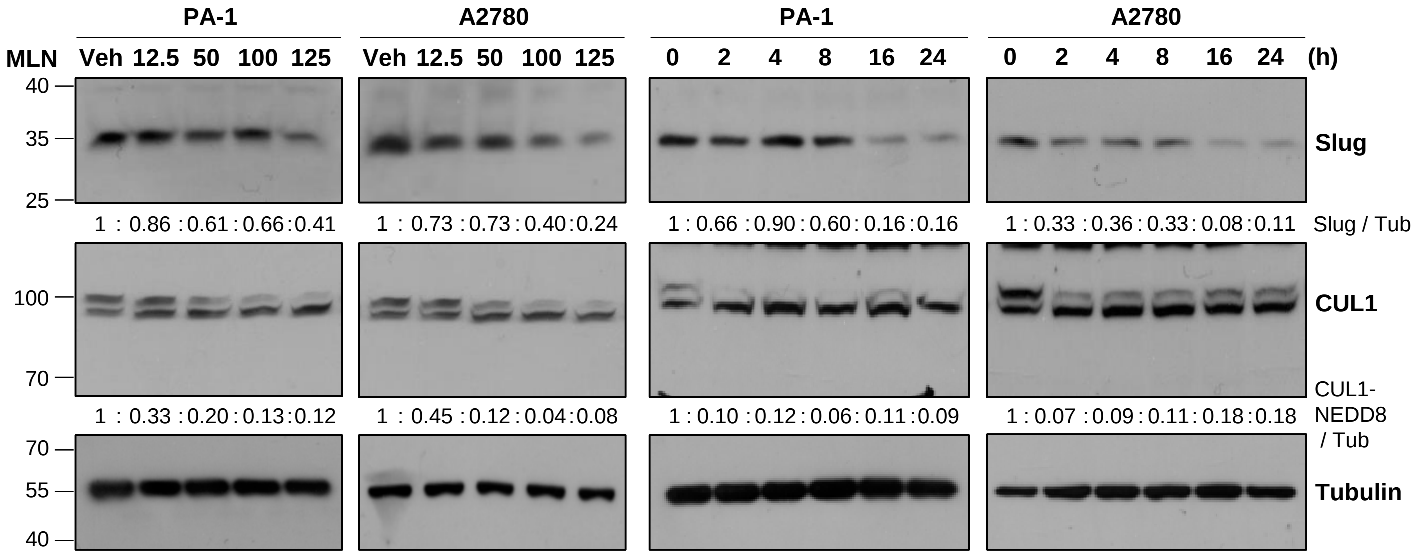

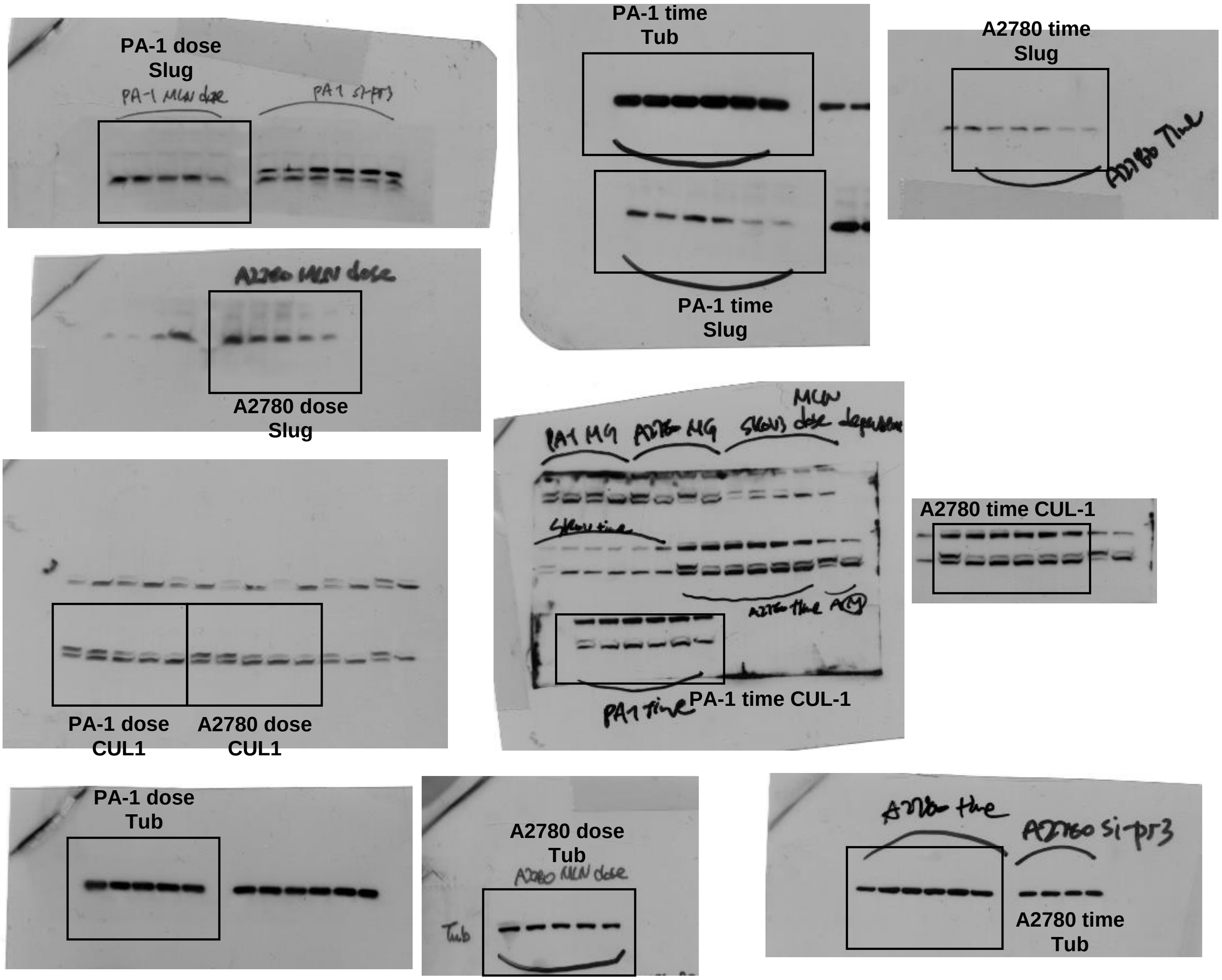

Original blots for western blot analysis shown in Figure 4A

Figure 4B, C

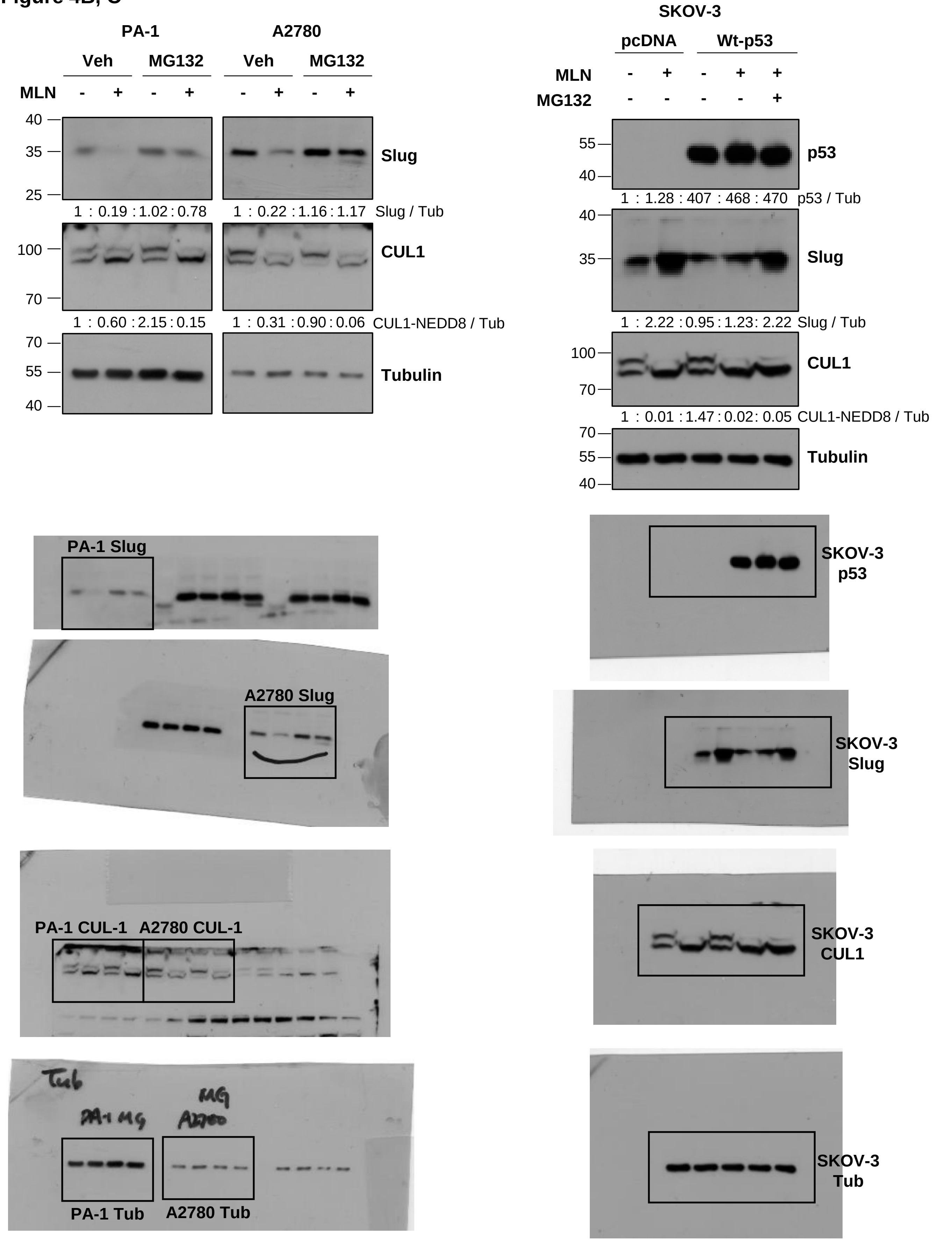

Original blots for western blot analysis shown in Figure 4B, C

Figure 4D

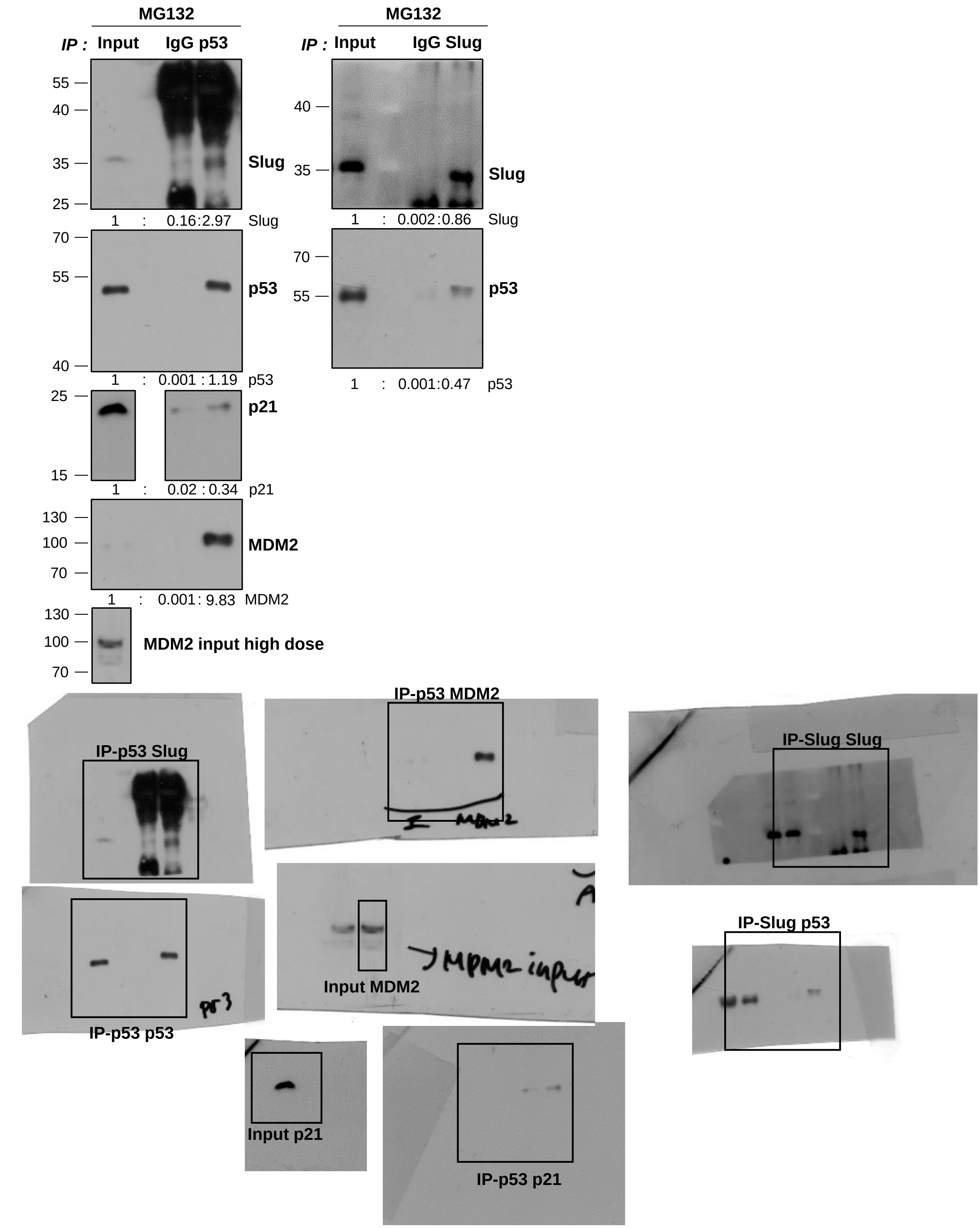

Original blots for western blot analysis shown in Figure 4D

Figure 4E

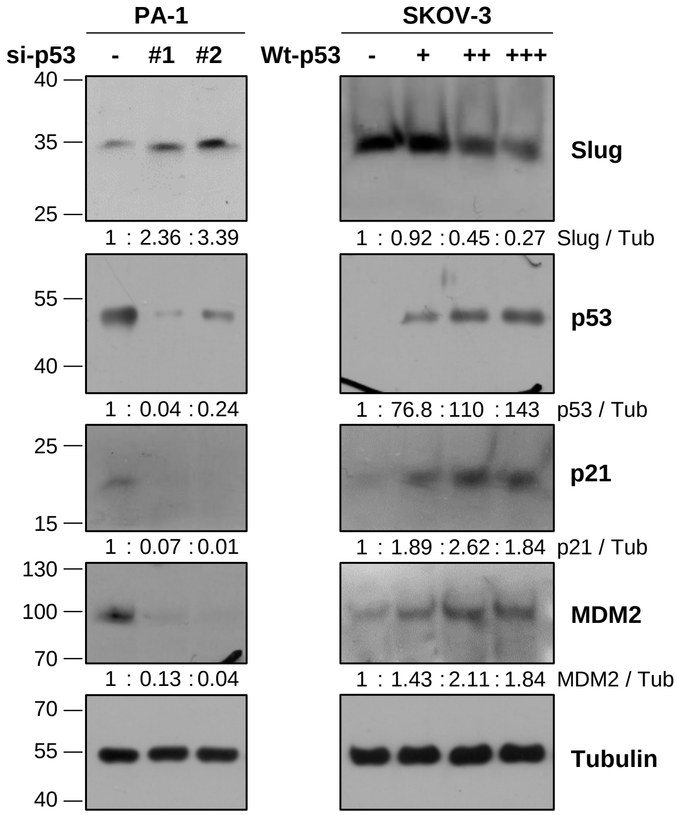

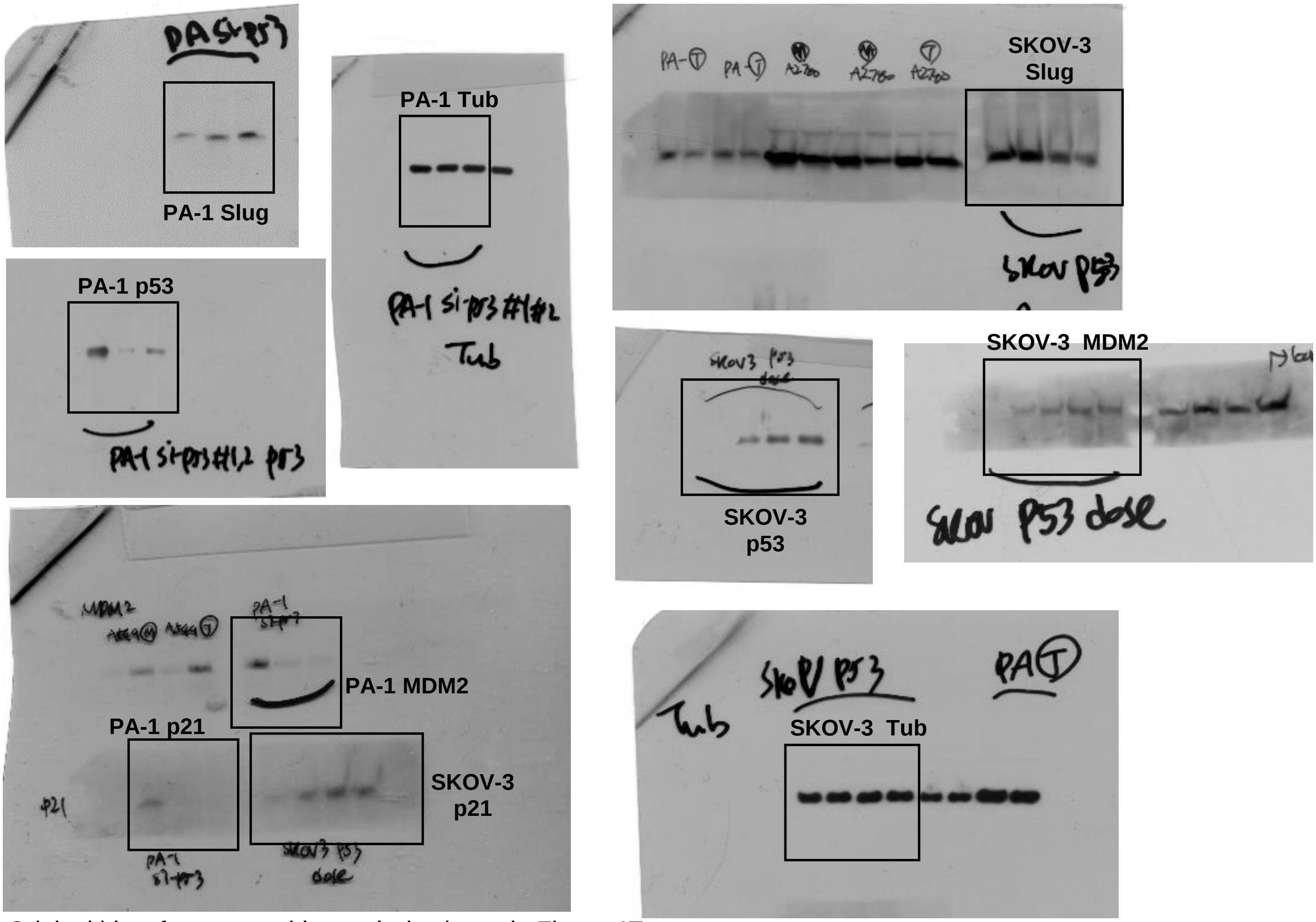

Original blots for western blot analysis shown in Figure 4E

Figure 4G

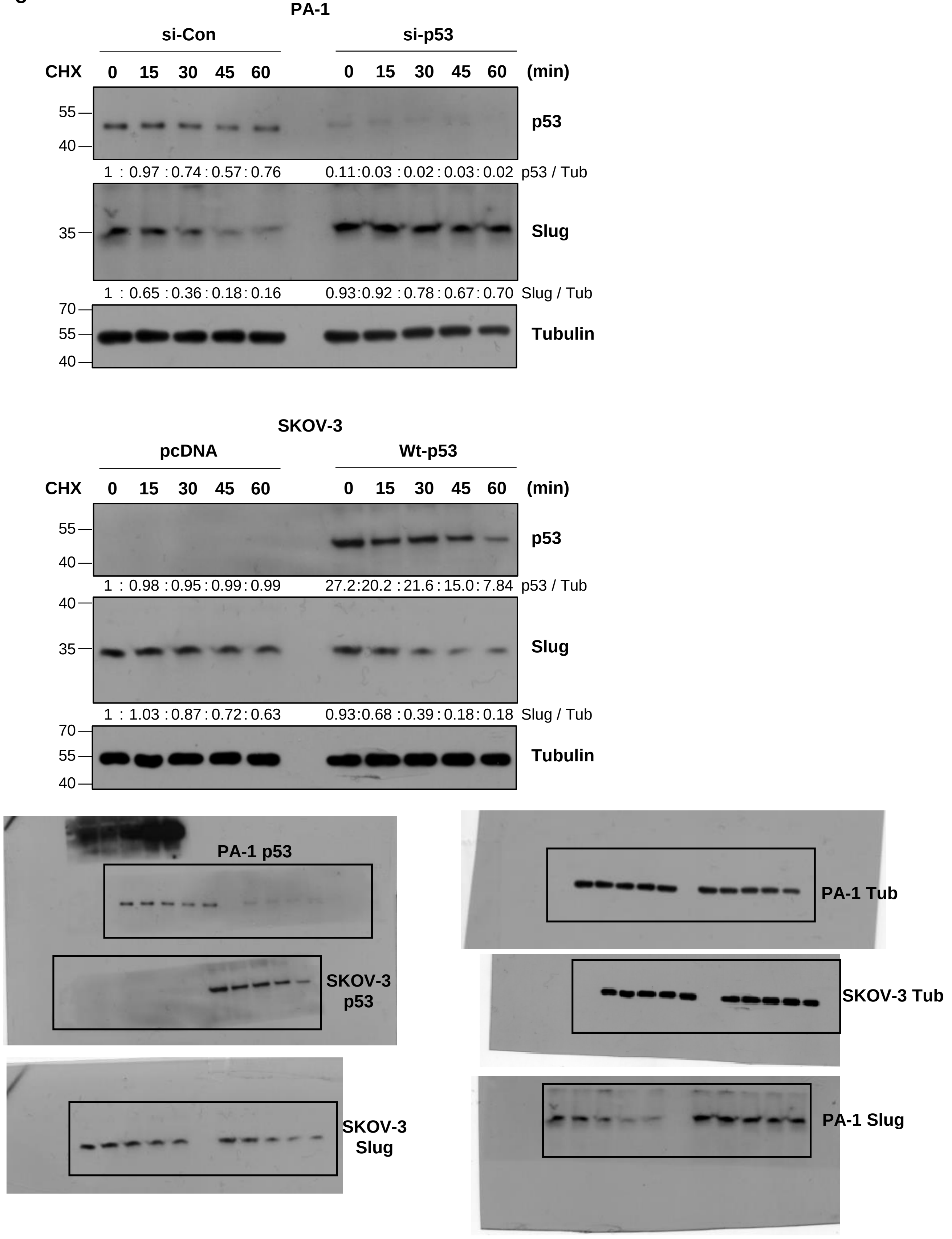

Original blots for western blot analysis shown in Figure 4G

Figure 4J

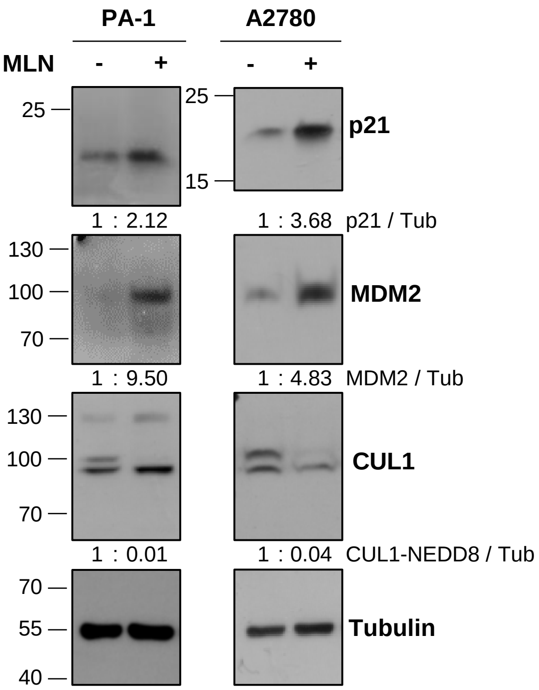

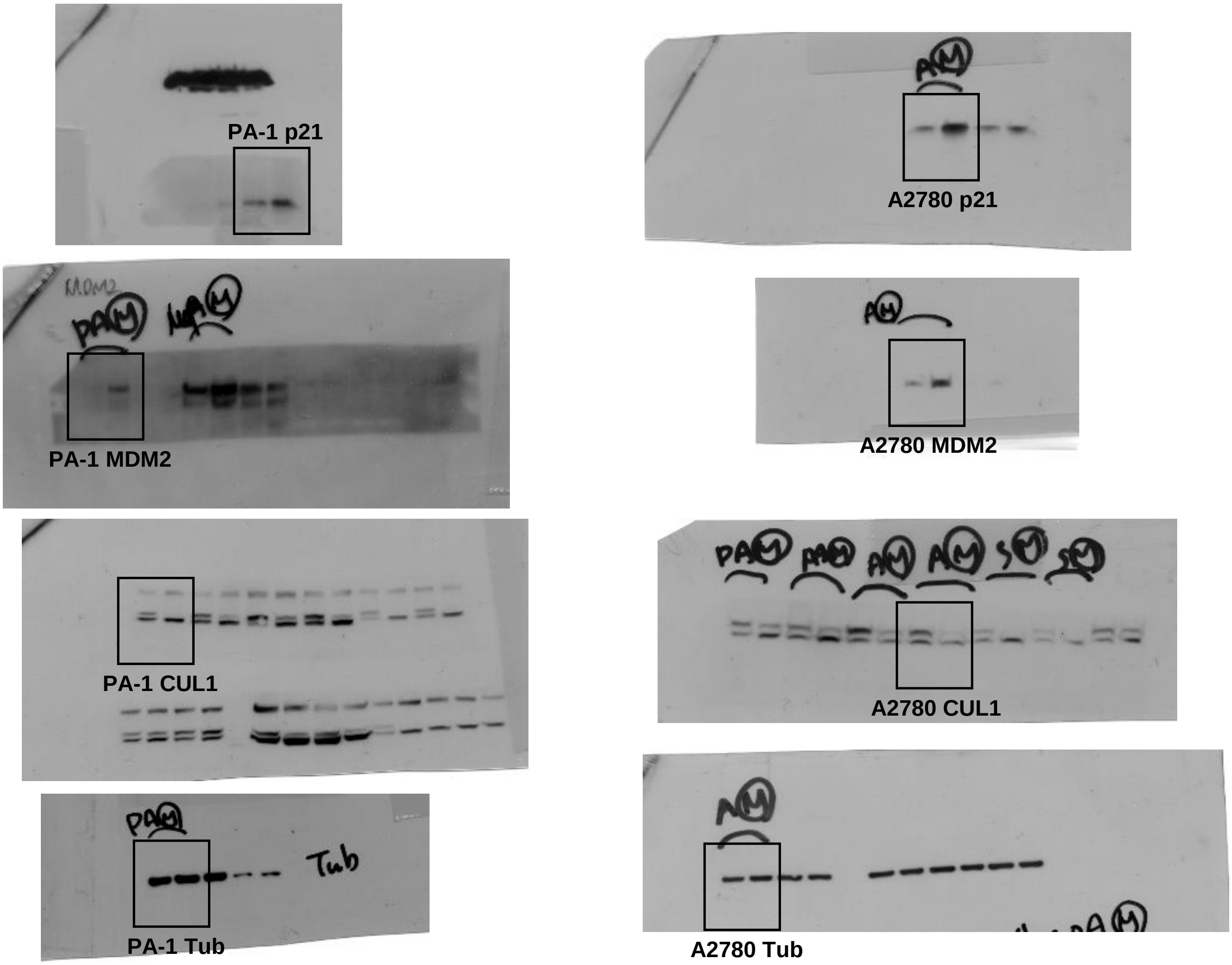

Original blots for western blot analysis shown in Figure 4J

Figure 4K

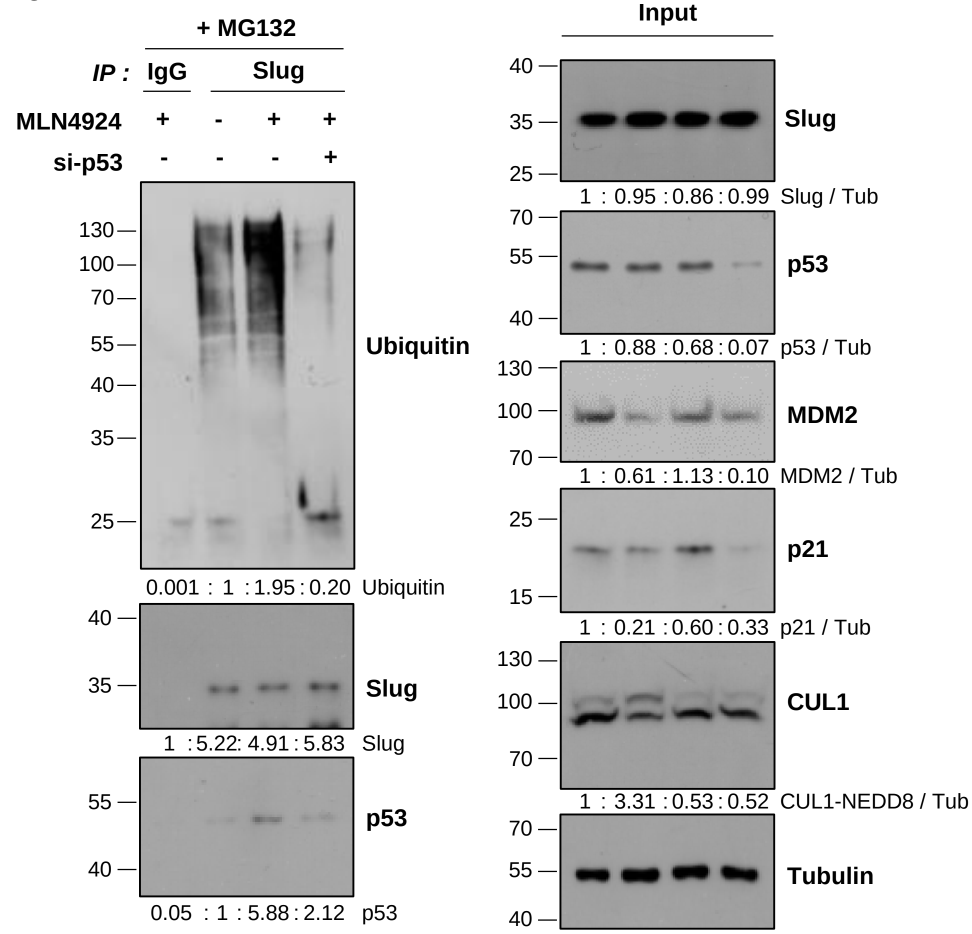

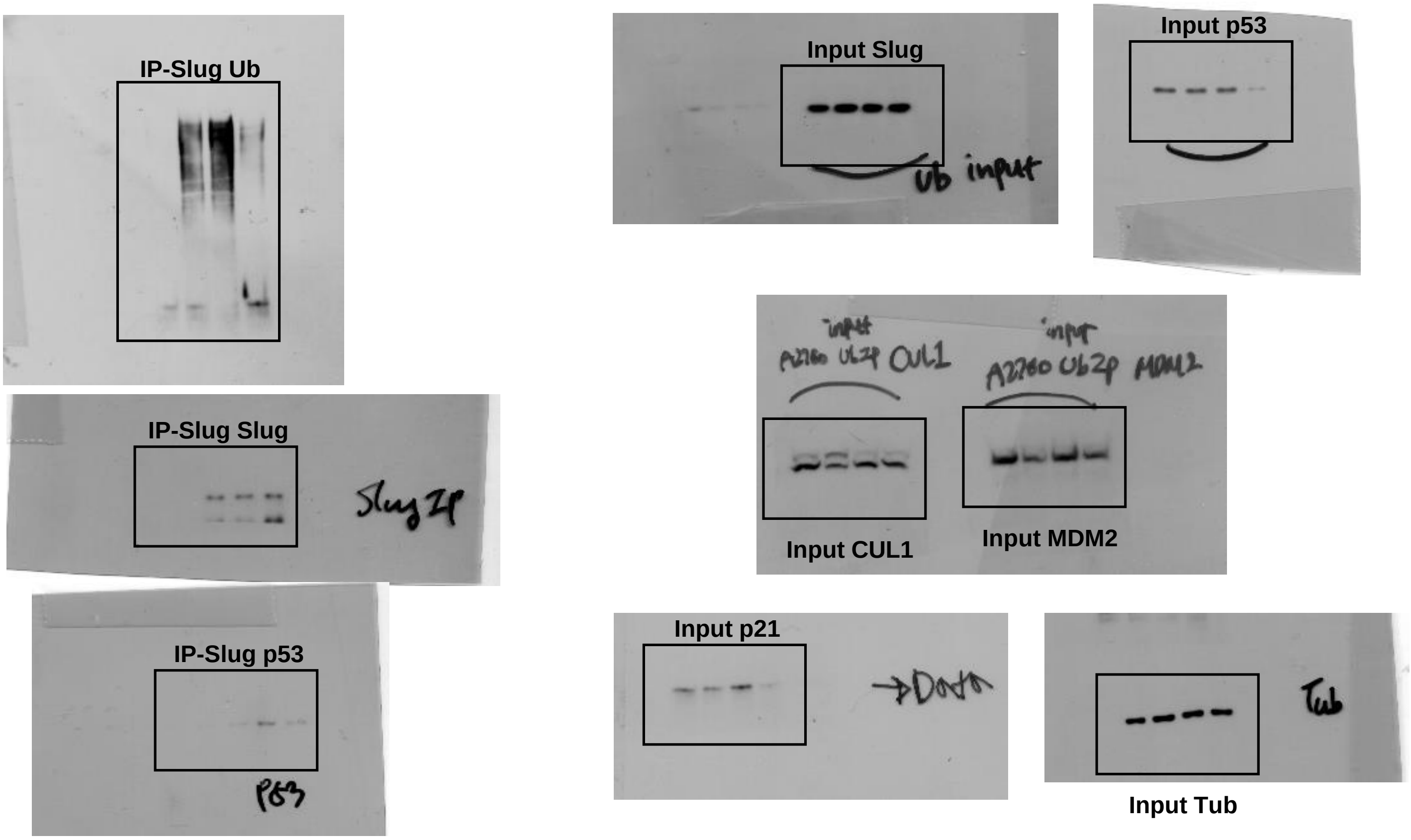

Original blots for western blot analysis shown in Figure 4K

Figure 5A

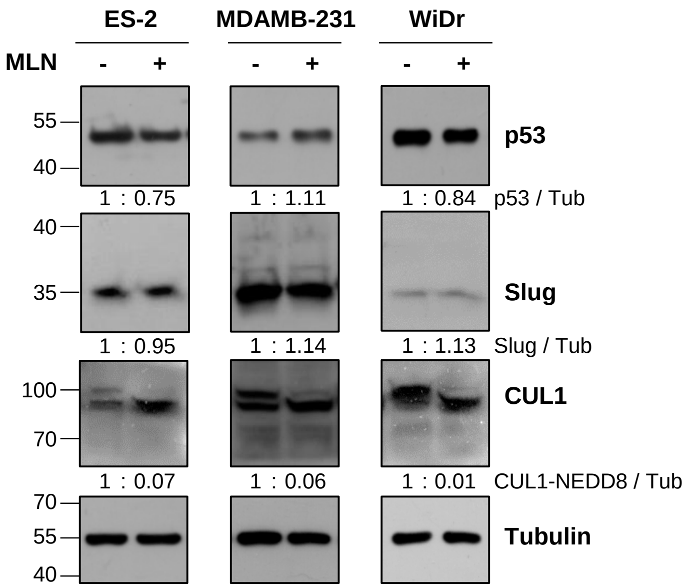

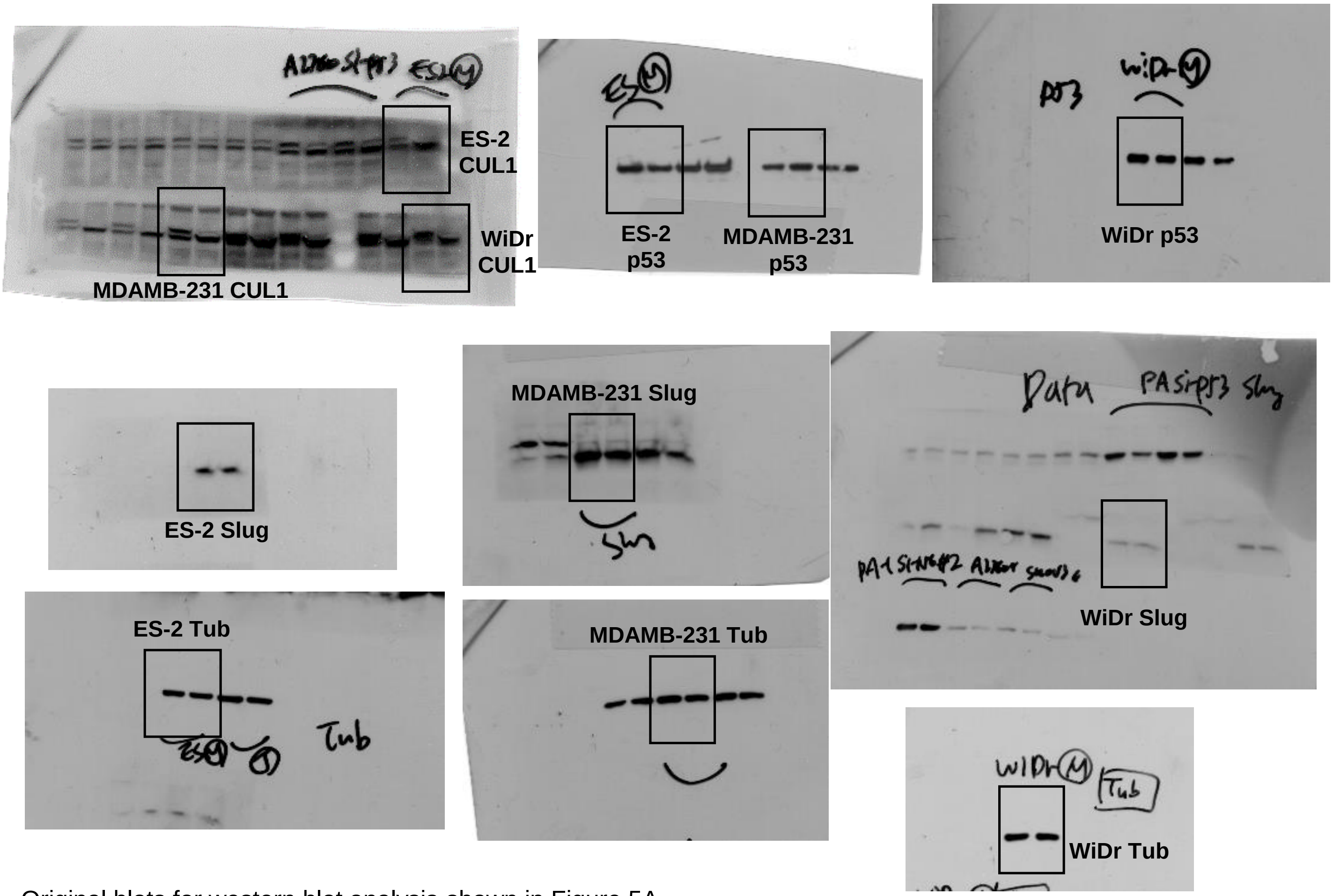

Original blots for western blot analysis shown in Figure 5A

Figure 5B

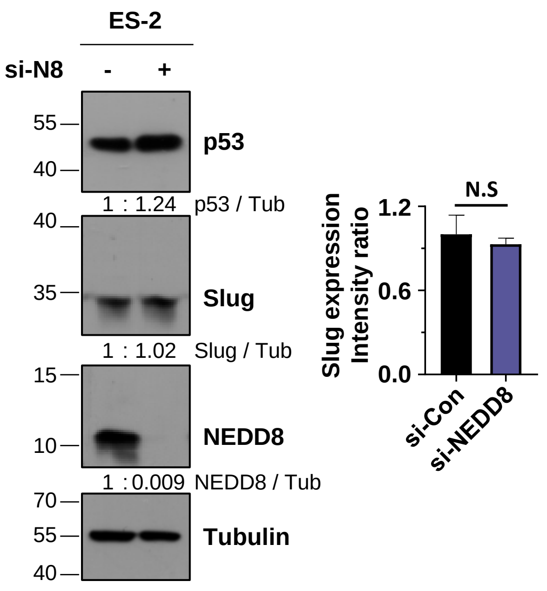

Figure 5G

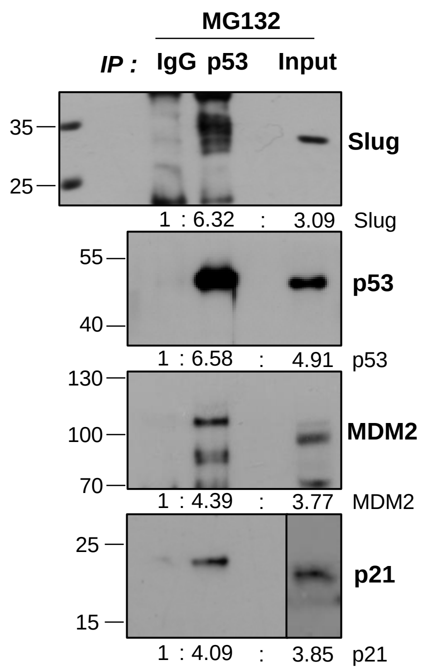

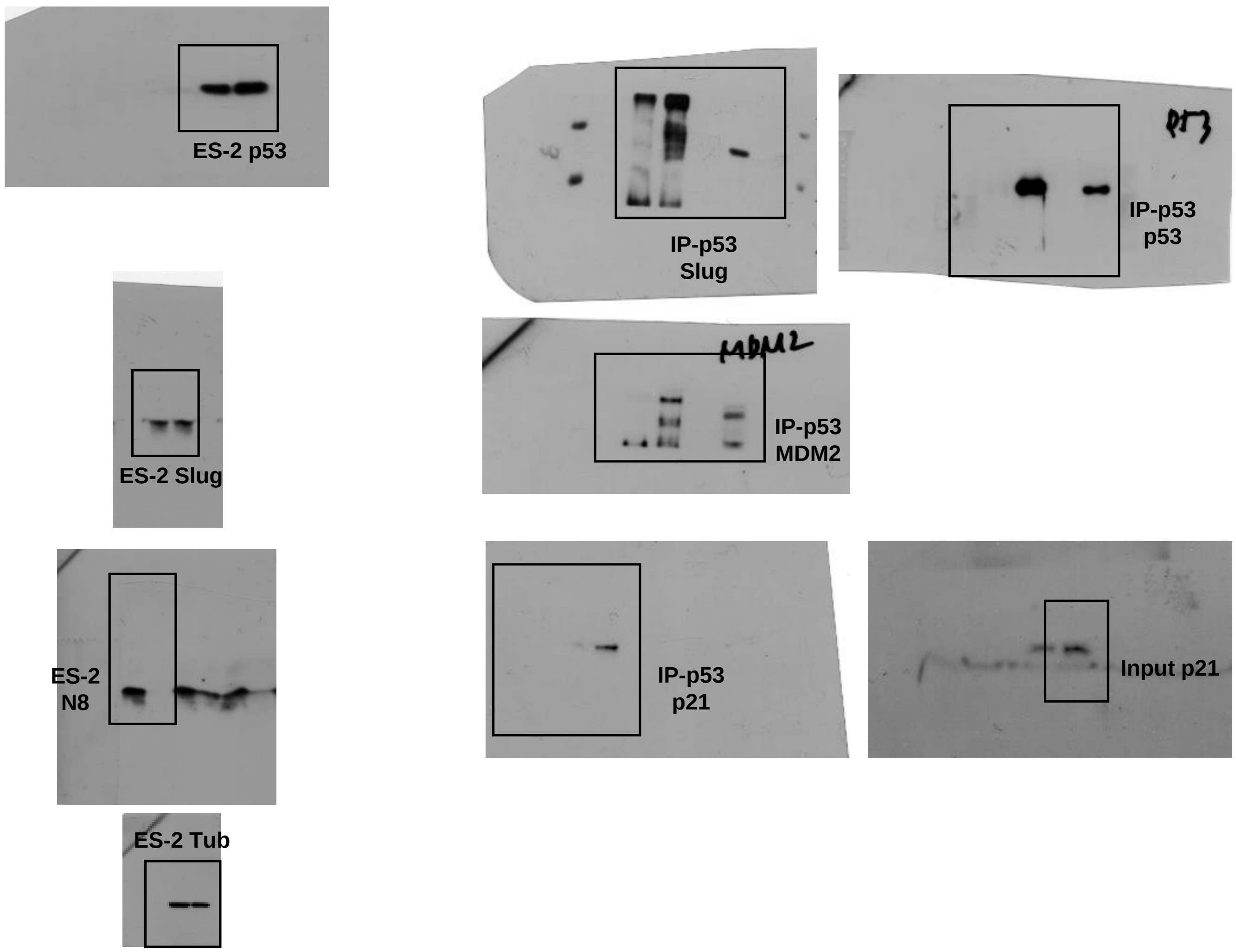

Original blots for western blot analysis shown in Figure 5B, G

Figure 5H

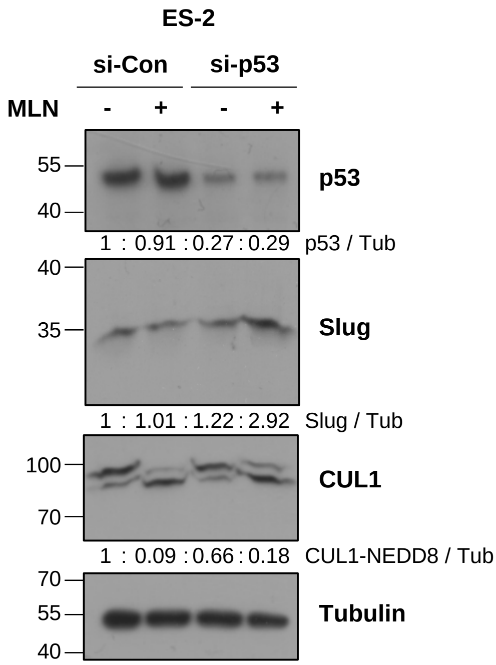

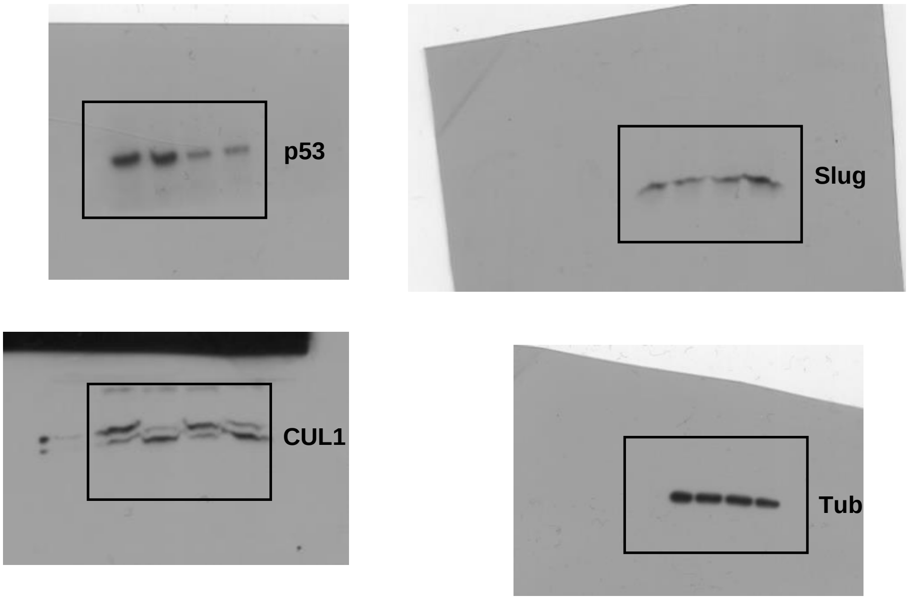

Original blots for western blot analysis shown in Figure 5H

Figure 6A

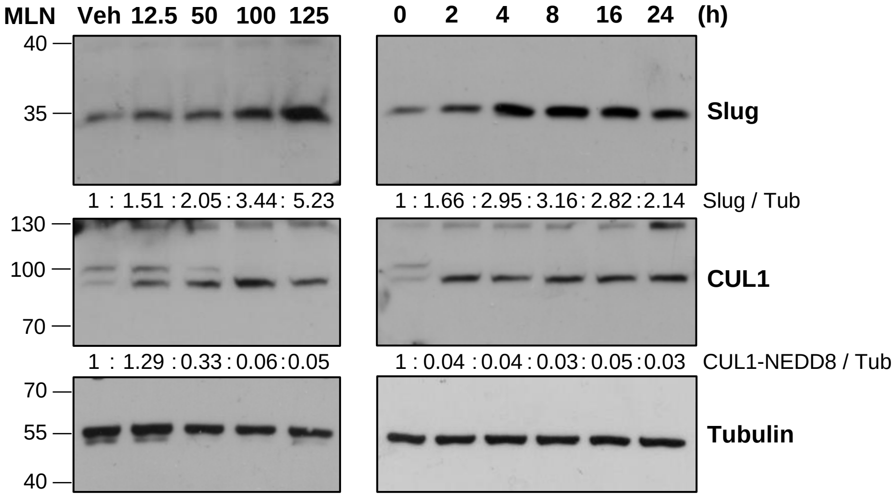

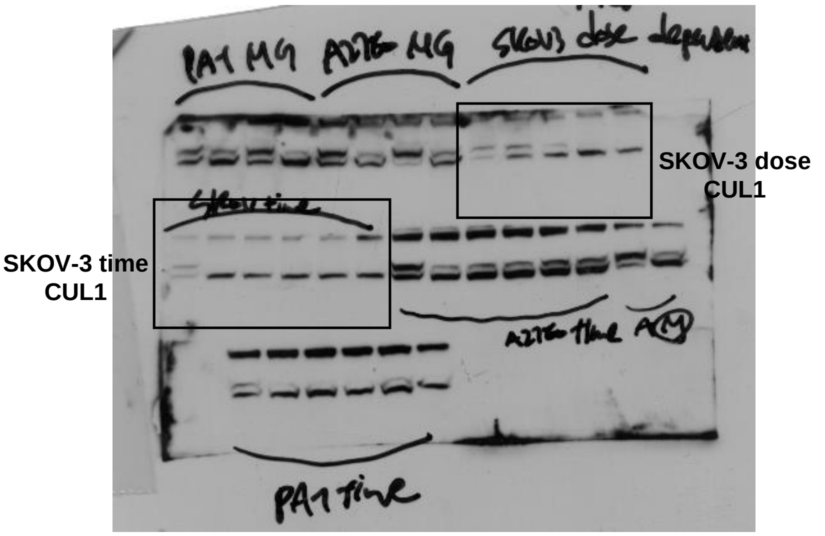

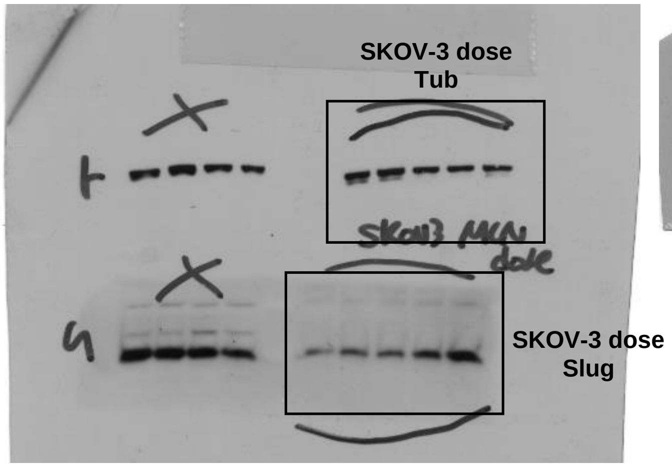

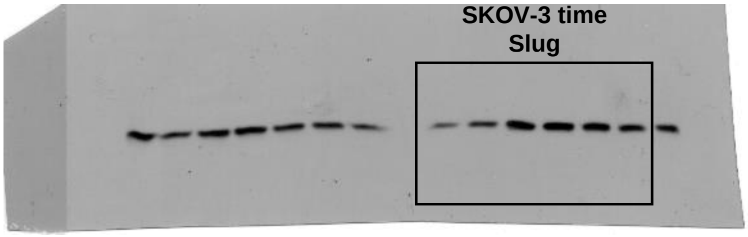

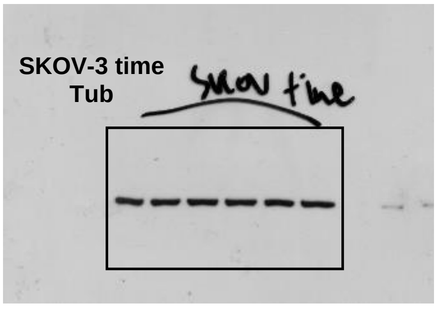

Original blots for western blot analysis shown in Figure 6A

Figure 6C

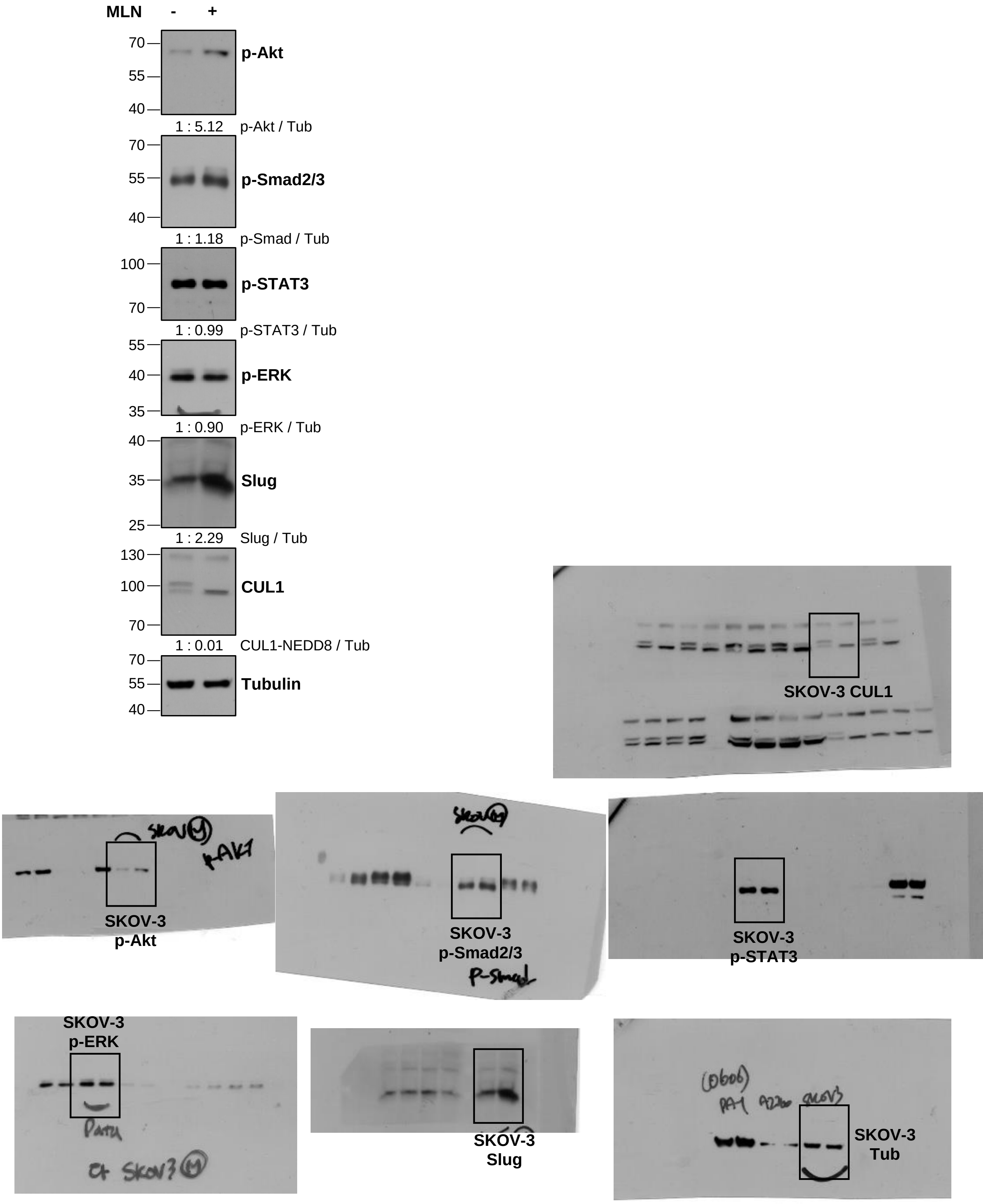

Original blots for western blot analysis shown in Figure 6C

Figure 6D

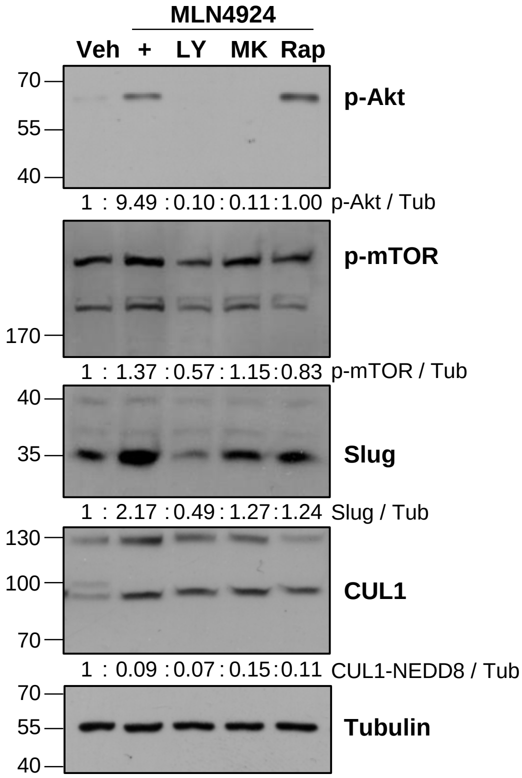

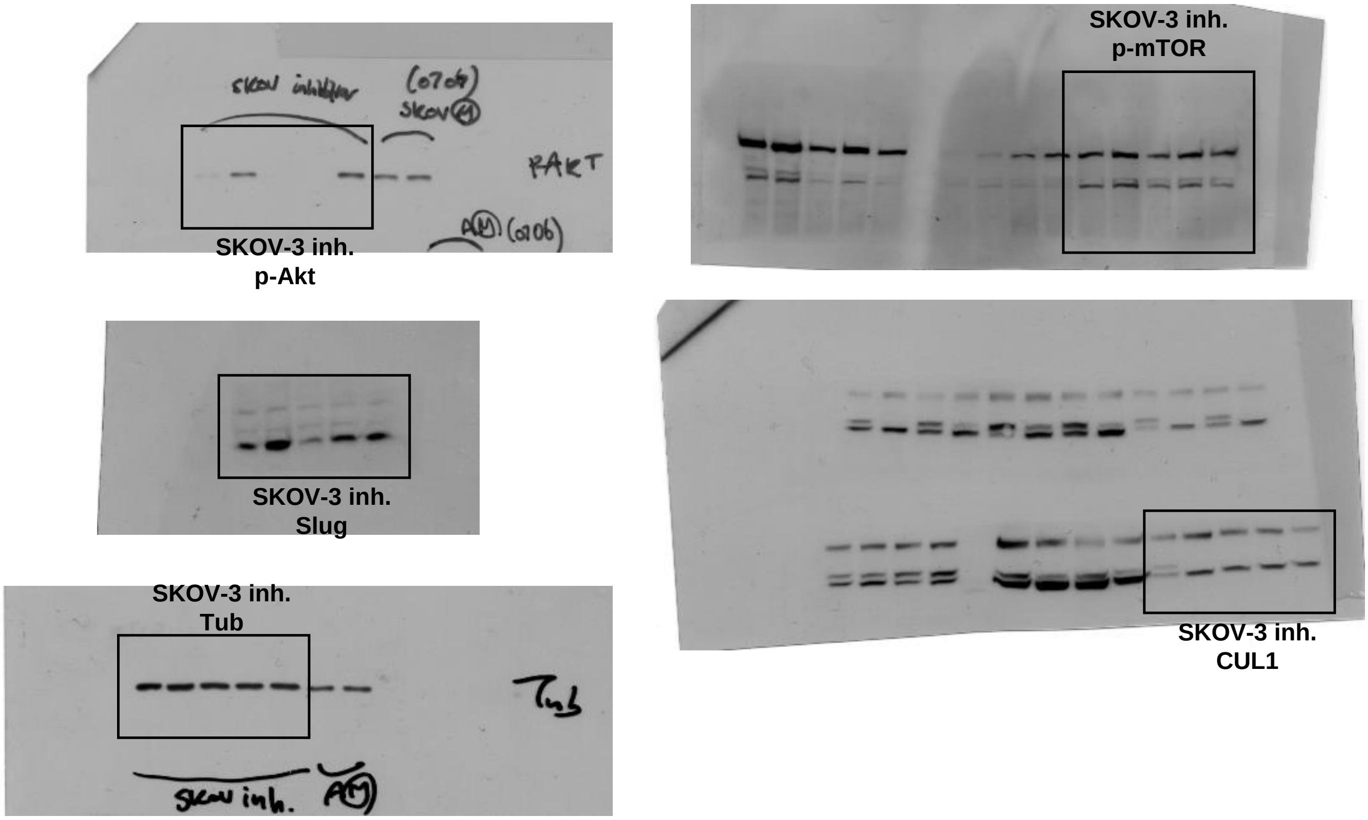

Original blots for western blot analysis shown in Figure 6D

Figure 7C

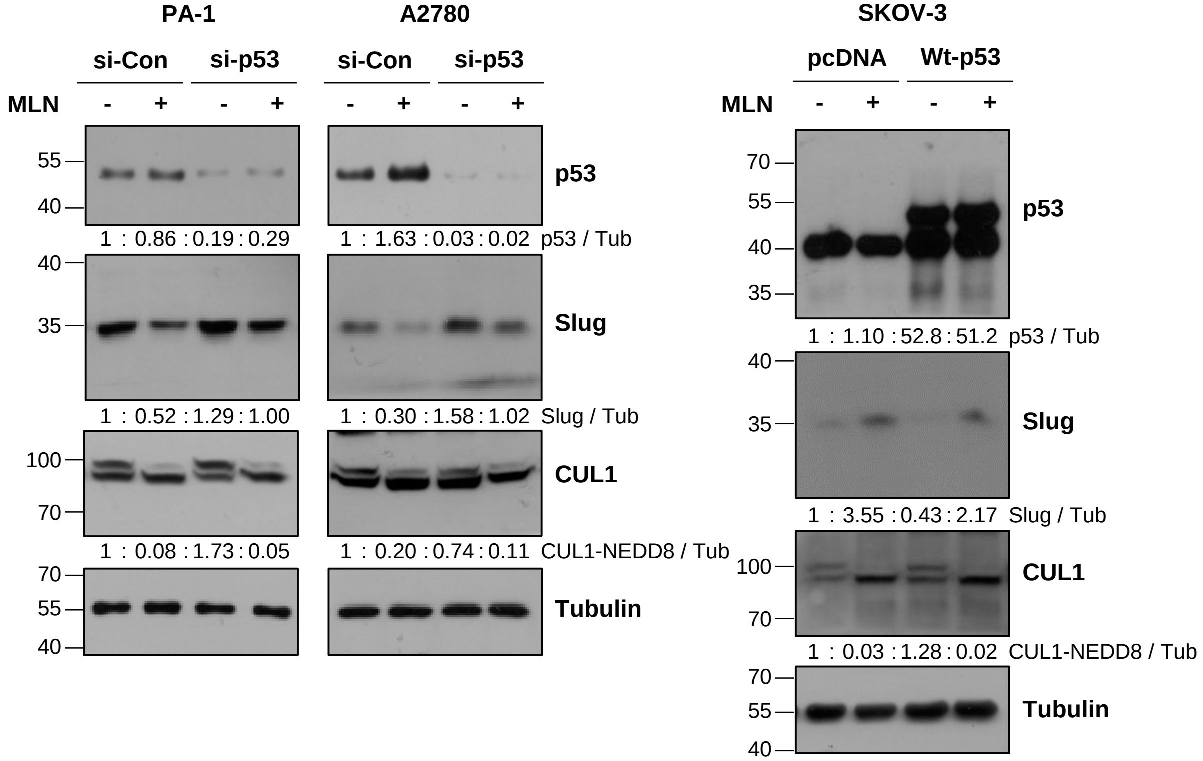

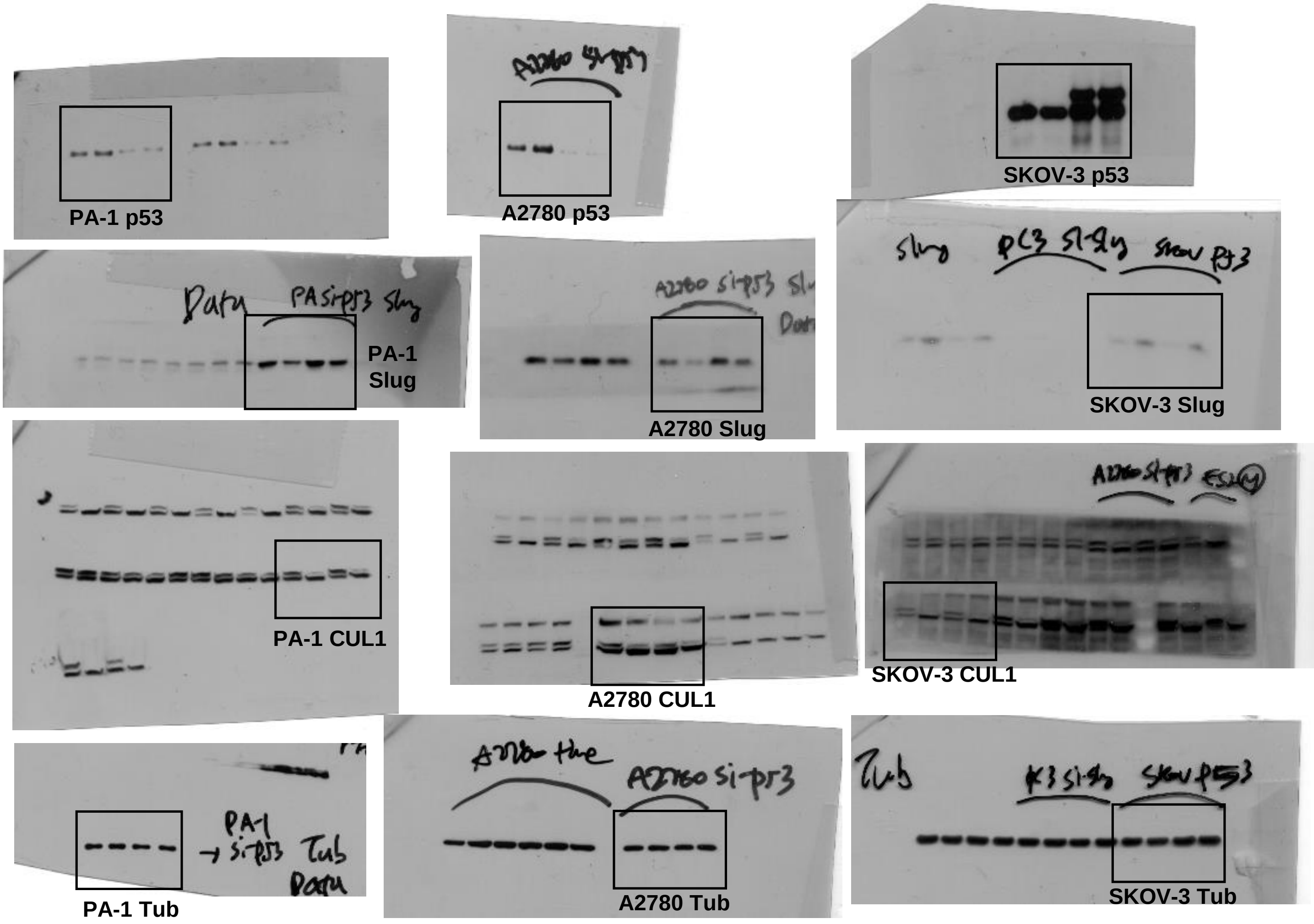

Original blots for western blot analysis shown in Figure 7C

Figure S2A

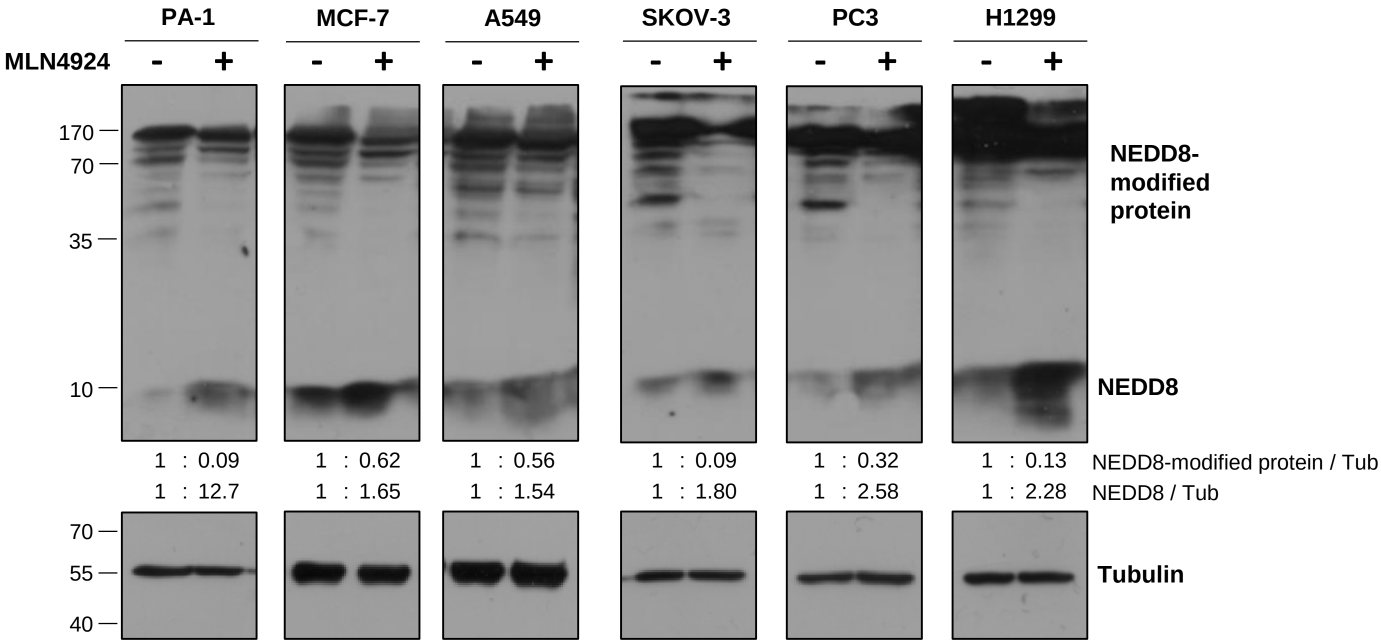

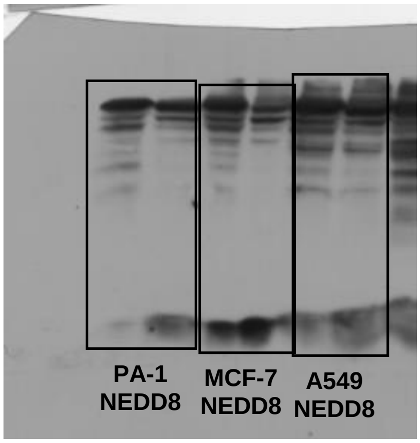

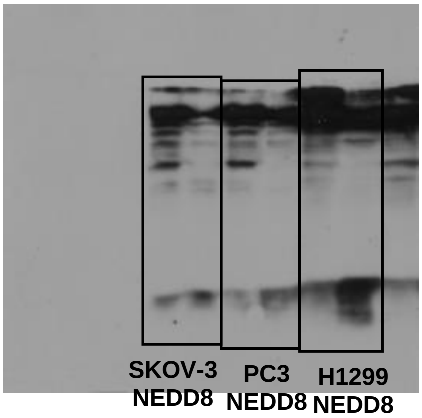

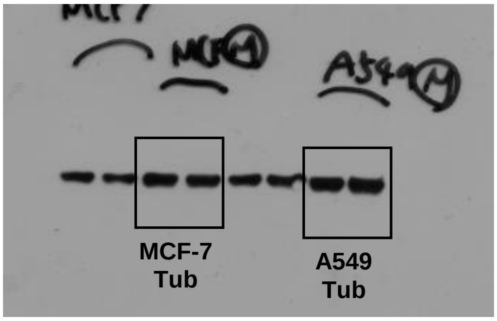

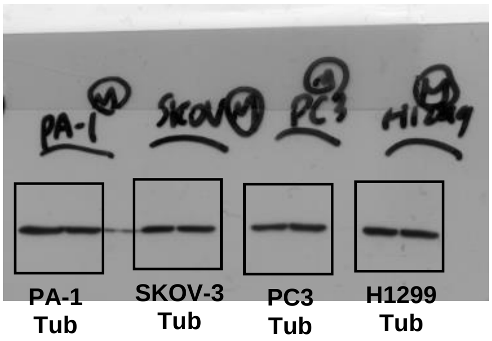

Original blots for western blot analysis shown in Figure S2A

Figure S3A

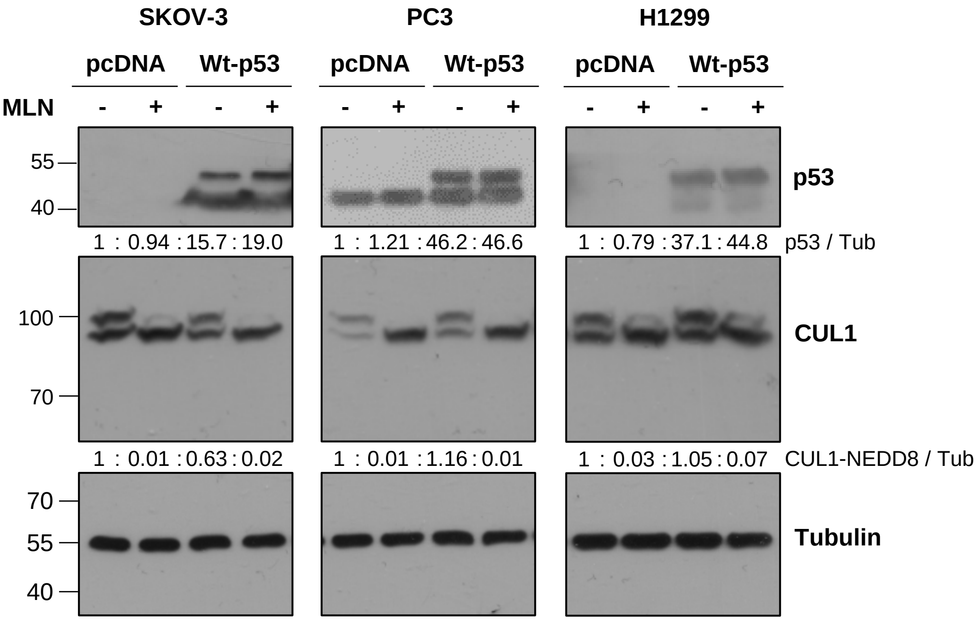

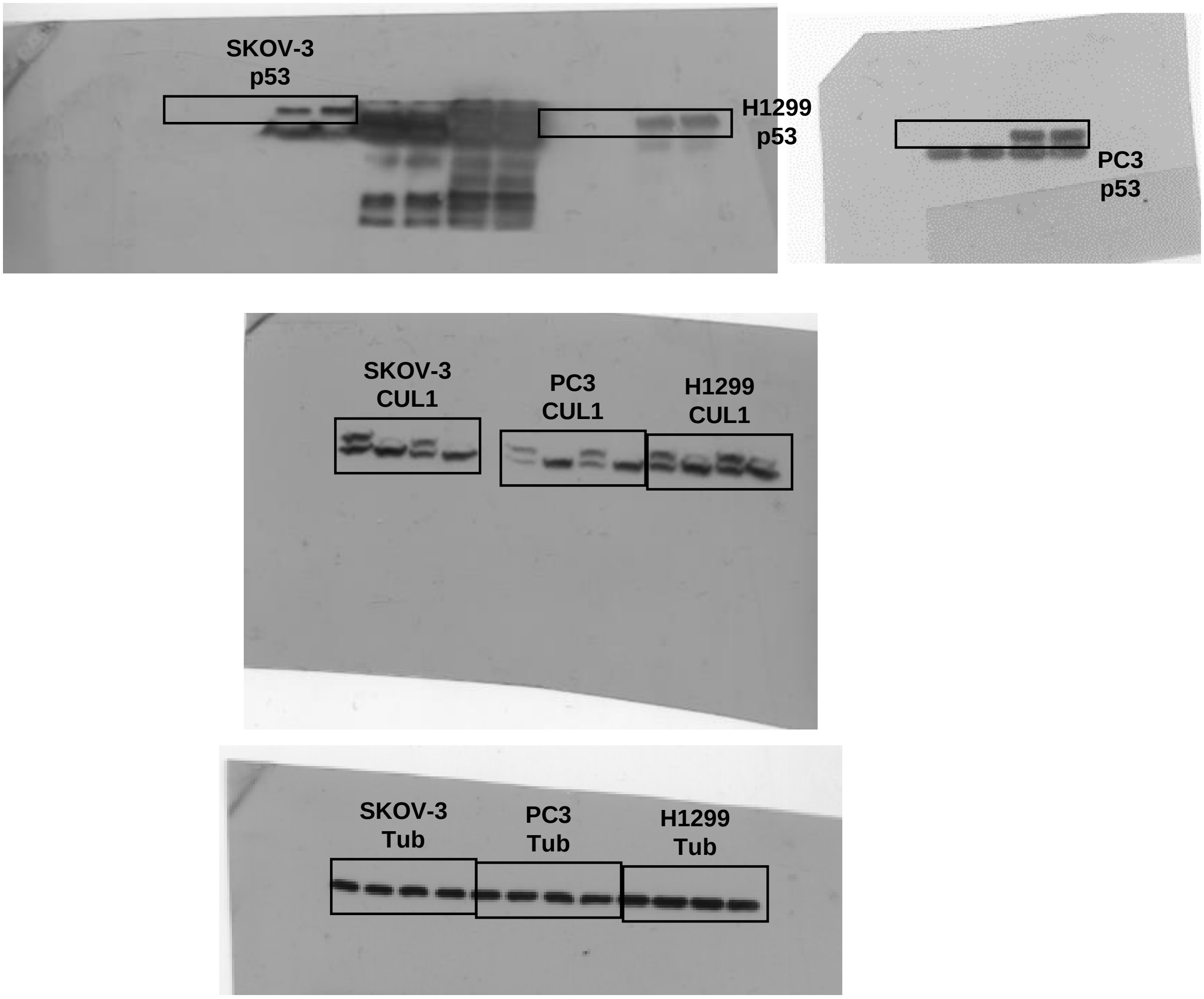

Original blots for western blot analysis shown in Figure S3A

Figure S4A

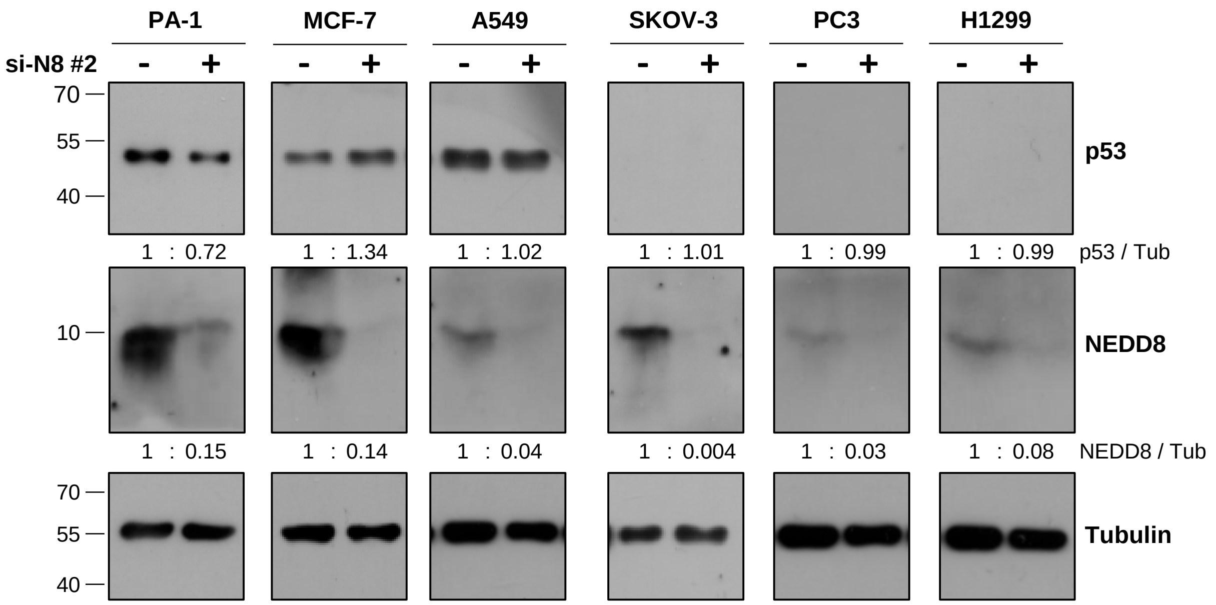

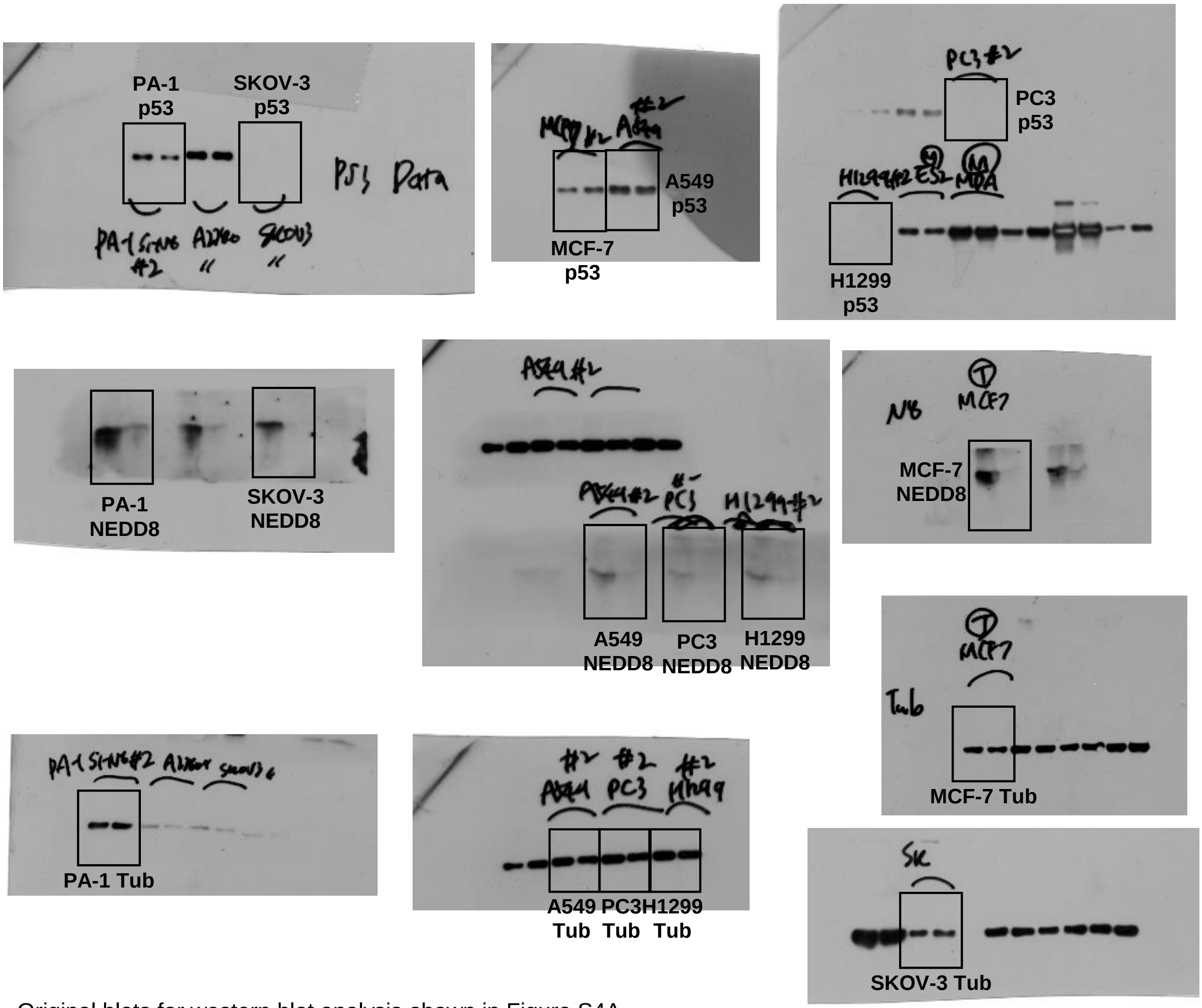

Original blots for western blot analysis shown in Figure S4A

Figure S5A

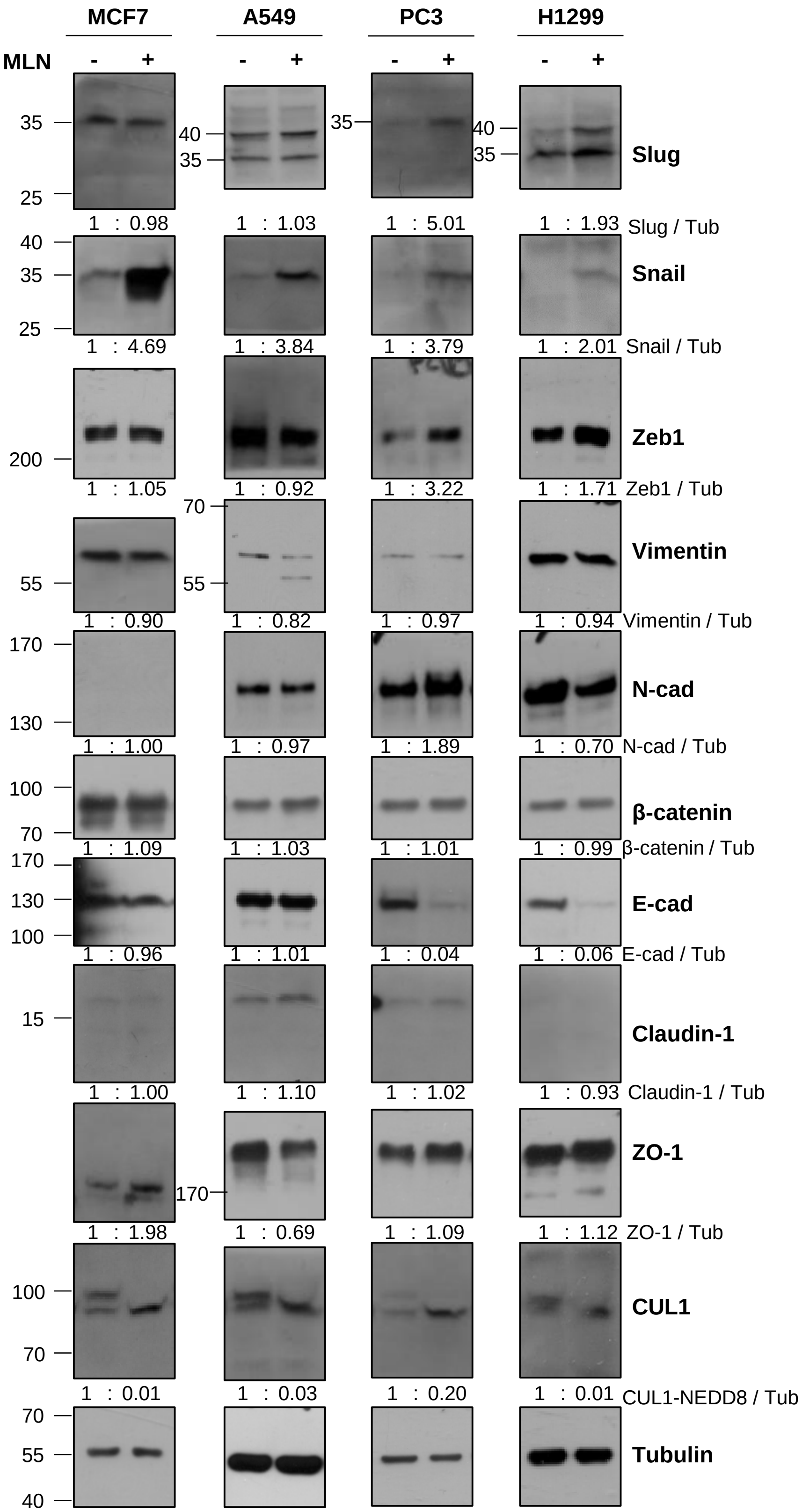

Original blots for western blot analysis shown in Figure S5A

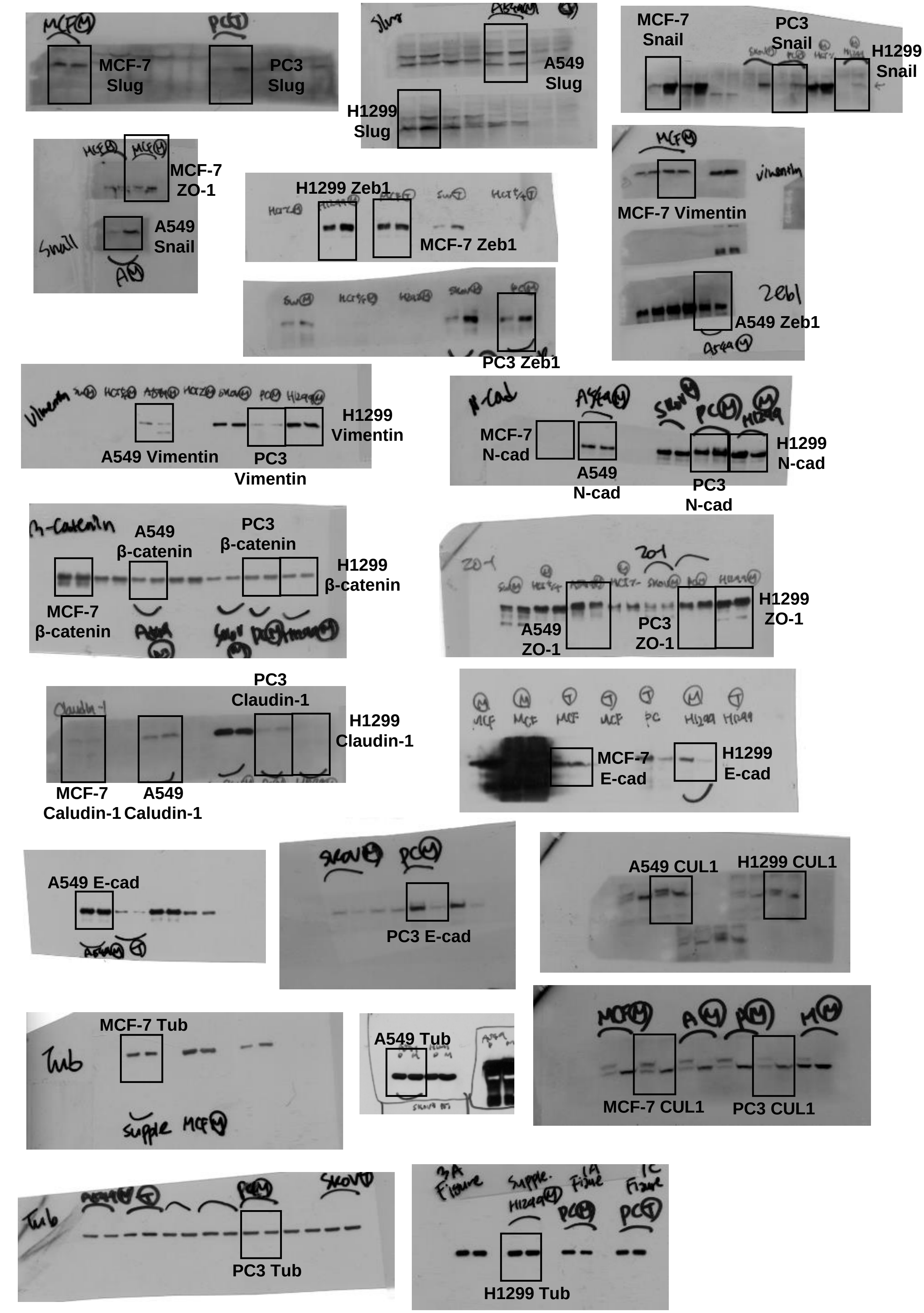

Original blots for western blot analysis shown in Figure S5A

Figure S5B

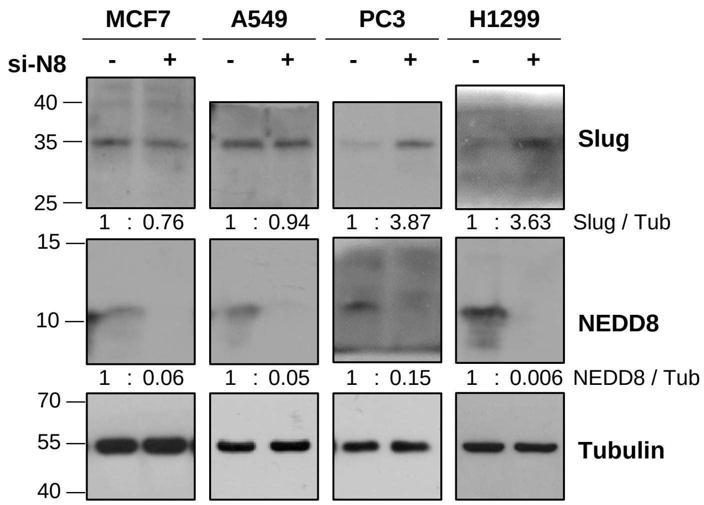

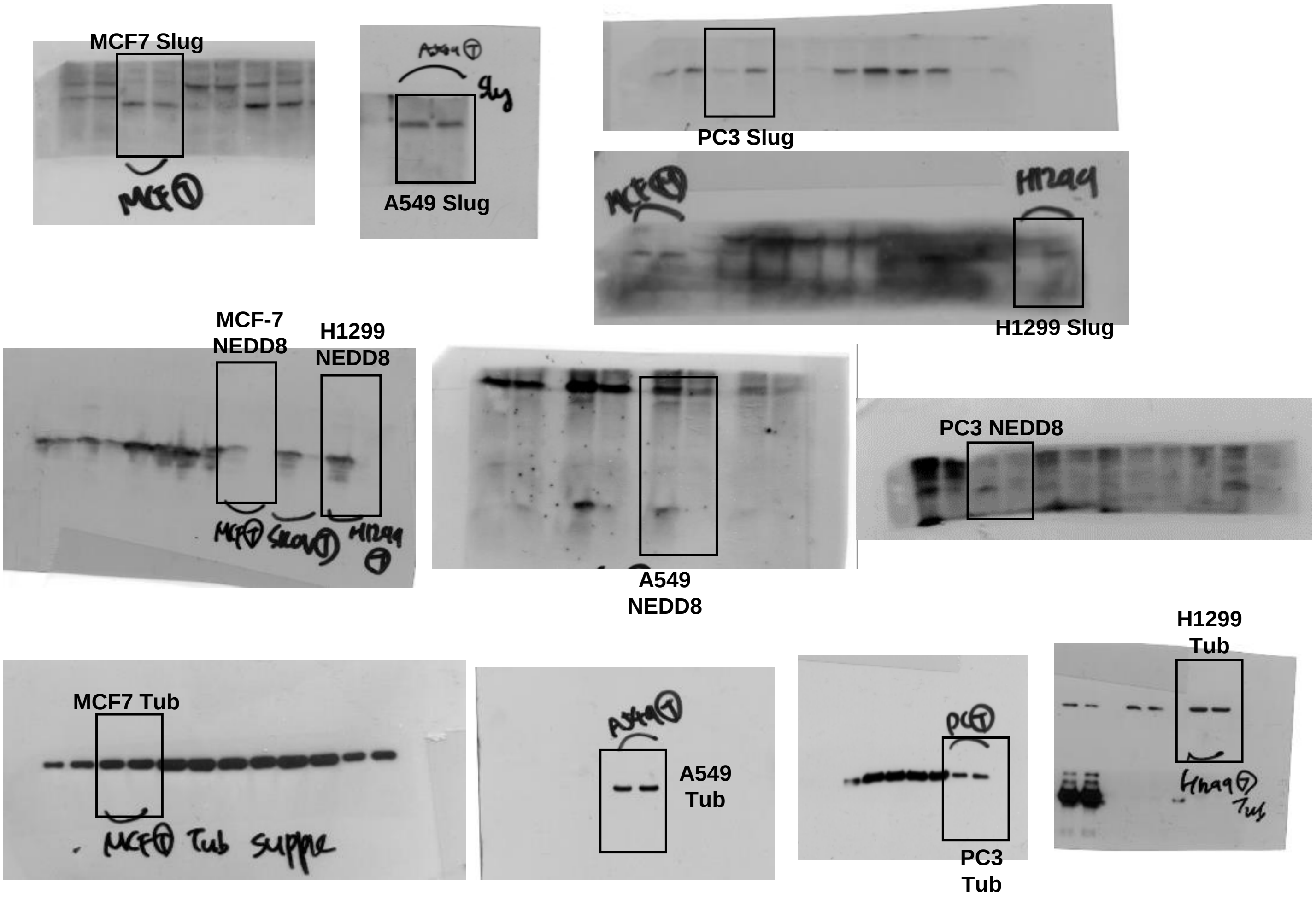

Original blots for western blot analysis shown in Figure S5B

Figure S6A

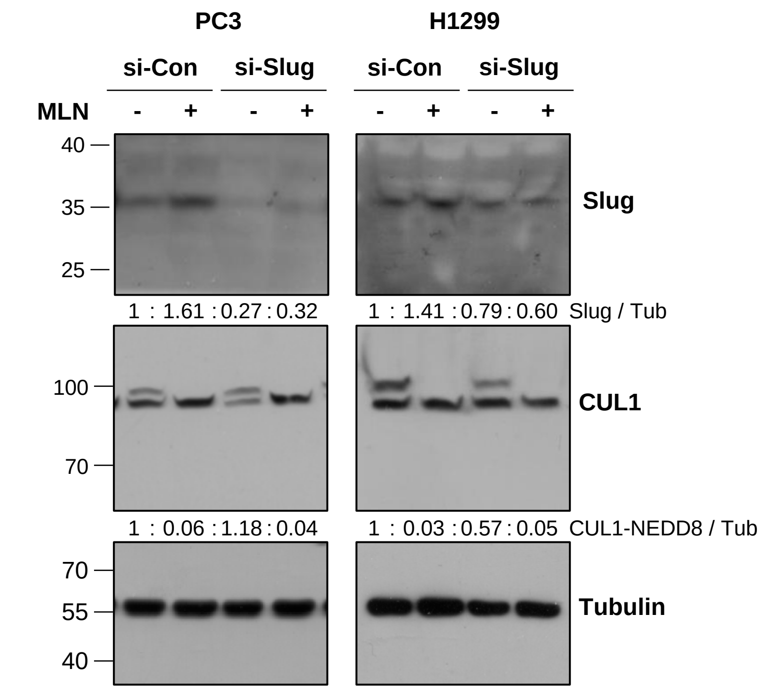

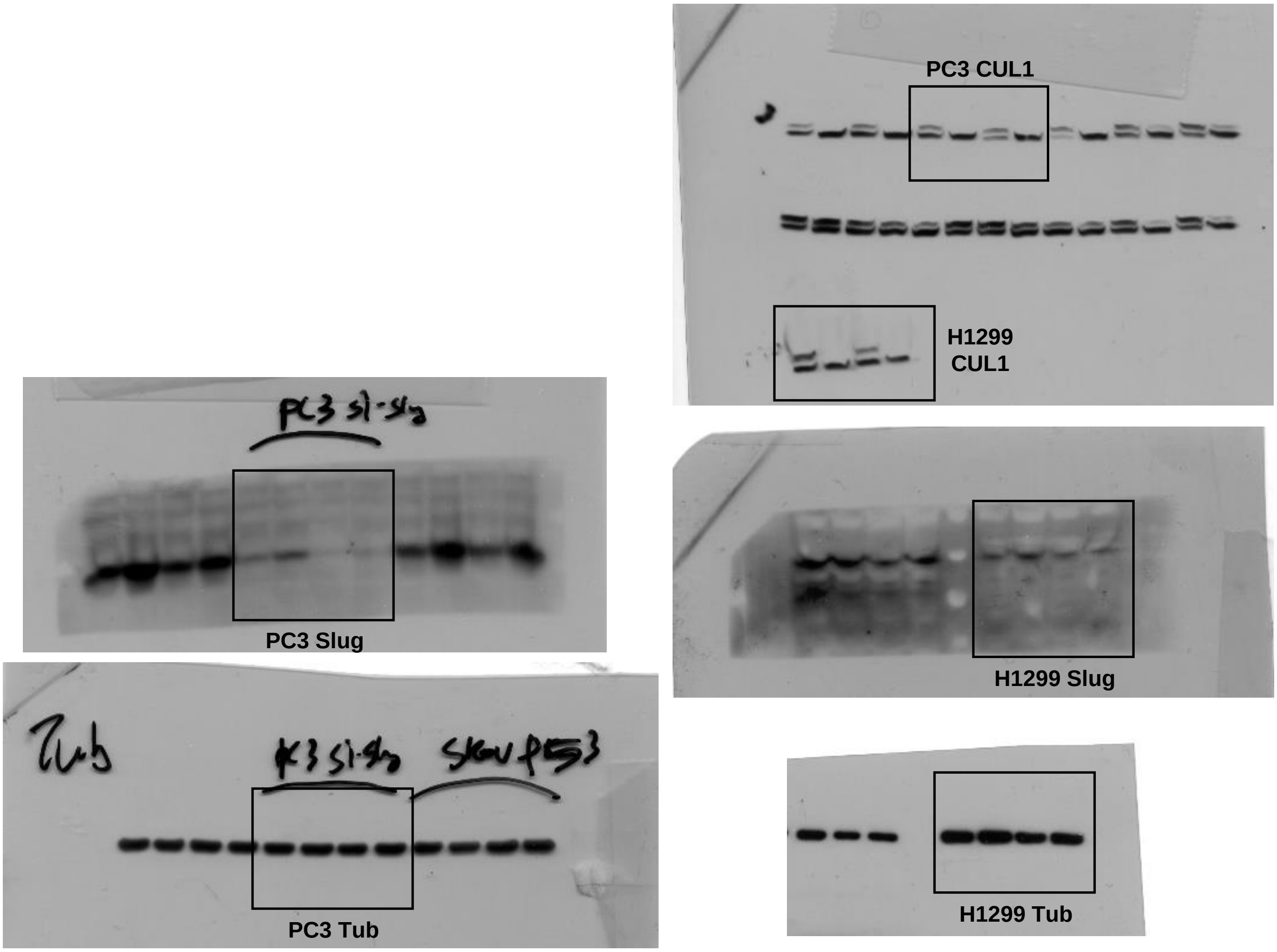

Original blots for western blot analysis shown in Figure S6A

Figure S8A

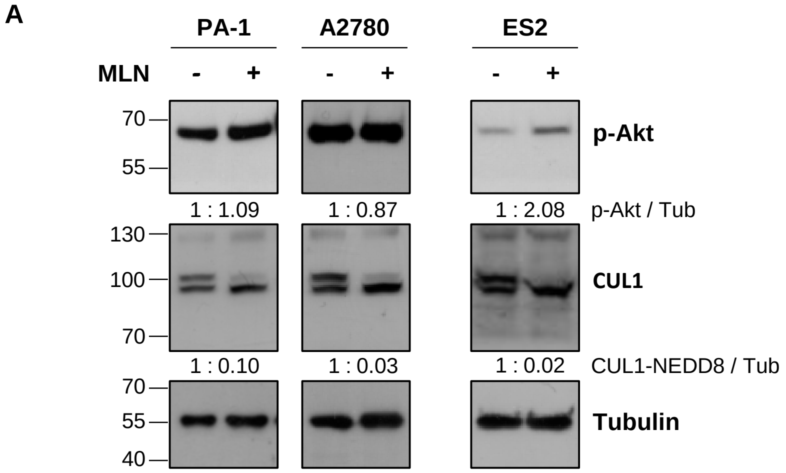

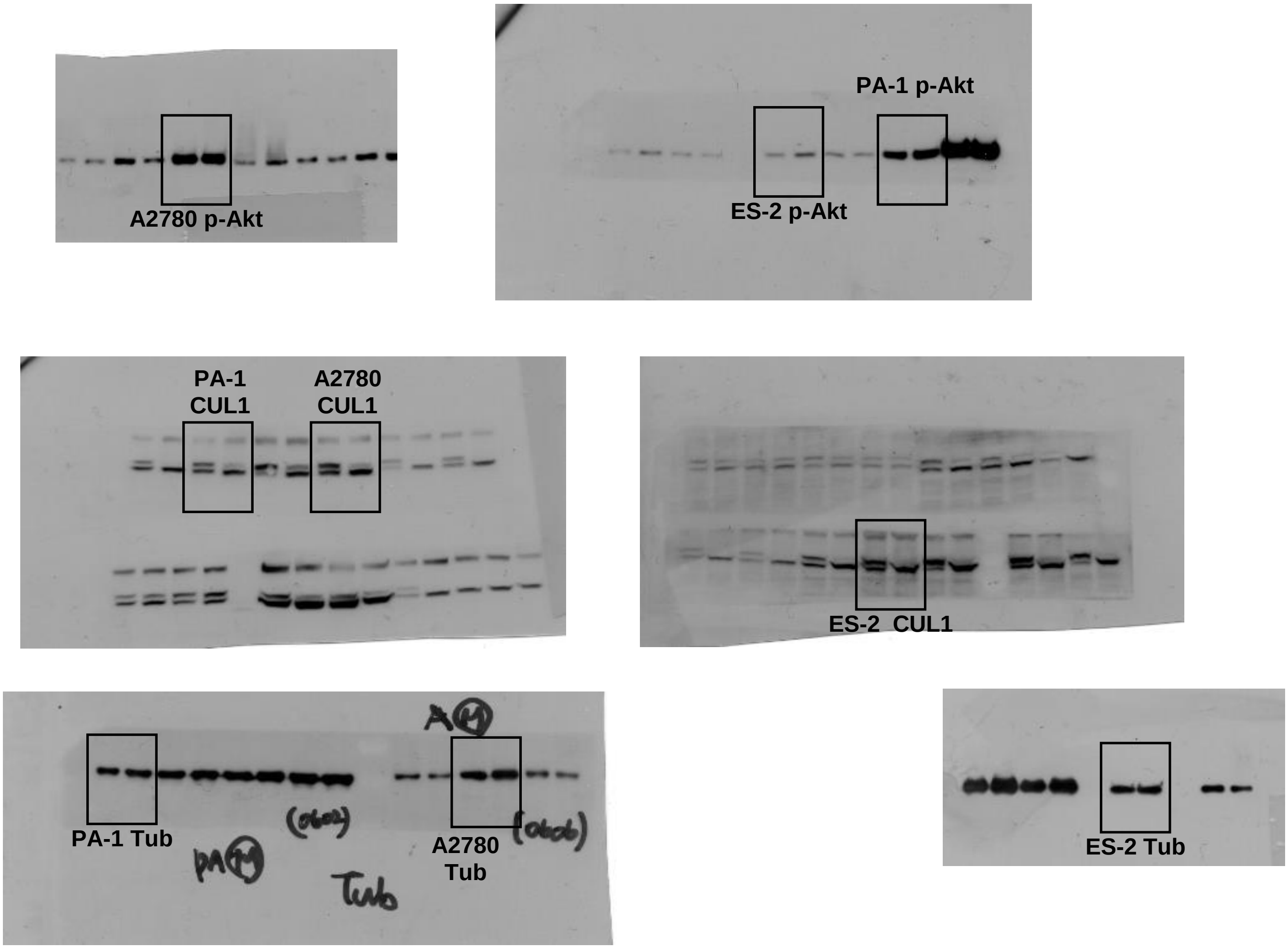

Original blots for western blot analysis shown in Figure S8A
